# Effects of Frying Temperature and Composite Spices on the Release Characteristics of Rapeseed Seasoning Oil

**DOI:** 10.3390/foods15040626

**Published:** 2026-02-09

**Authors:** Ailikemu Mulati, Yuting Yang, Xinmeng Huang, Yuanpeng Li, Aihemaitijiang Aihaiti, Jing Lu, Yuanyuan Hou, Jiayi Wang

**Affiliations:** College of Smart Agriculture (Research Institute), Xinjiang Key Laboratory of Biological Resources and Genetic Engineering, Xinjiang University, Urumqi 830046, China

**Keywords:** spice, flavored oil, volatile organic compounds, frying temperature

## Abstract

In Chinese cuisine, seasoning oil enhances the aroma and appearance of dishes. This study examined how processing affects flavor release in multi-ingredient oils. Volatile organic compounds (VOCs), relative odor activity value (ROAV), and variable importance projection (VIP) were used to assess flavor changes. Optimal frying was 160 °C for 15 min with 11% green Sichuan peppercorn, 3% ghost pepper, 6% green onion, 0.1% bay leaf, 0.2% deseeded tsaoko, 0.5% star anise, 0.3% fennel seeds, 1.5% dried Erjingtiao chili, 5% ginger, and 2.5% red Sichuan peppercorn. Gas chromatography–ion mobility spectrometry (GC-IMS) and gas chromatography–mass spectrometry (GC-MS) analyzed heating at 150 °C, 160 °C, and 170 °C. Temperature strongly influenced VOC formation; 160 °C produced the most diverse VOCs, including aldehydes, ketones, terpenes, esters, and alcohols. Multivariate analysis identified 73 key compounds (VIP > 1) between 150 and 160 °C, but only 39 between 160 and 170 °C, indicating that high heat reduces complexity. Compounds such as 2-methylpyrazine and (E)-2-heptenal contributed caramel, nutty, buttery notes, with 2-methoxy-3-(1-methylethyl)-pyrazine as the core aroma. Frying at 160 °C balanced sweet, floral, and roasted aromas, offering guidance for precise seasoning oil flavor control.

## 1. Introduction

Seasoning oil is produced from edible vegetable oil through the thermal extraction of aromatic compounds from plant tissues, a process that facilitates the dissolution of lipophilic bioactives into the lipid base to enhance the aroma, luster, and flavor complexity of Chinese cold and stir-fried dishes [1]. Furthermore, the addition of spices imparts distinct sensory properties, including fragrance, freshness, sweetness, spiciness, and numbing sensations [2]. These botanical ingredients also serve as rich reservoirs of bioactive compounds, exemplified by complex matrices such as green and red Sichuan peppercorns (*Zanthoxylum schinifolium* and Z. *bungeanum*), known for sanshool-induced paresthesia and volatile terpenes [3]. Ghost pepper (*Capsicum chinense*) and dried Erjingtiao chili, which contribute varying degrees of capsaicinoid-driven pungency and carotenoid pigments [4]. ginger (Zingiber officinale) and green onion, providing sulfur-containing volatiles and pungent gingerols [5]; and star anise (Illicium verum), fennel seeds (Foeniculum vulgare), bay leaf (Laurus nobilis), and deseeded tsaoko (Amomum tsaoko). Collectively, these ingredients are characterized by high concentrations of polyphenols and flavonoids that contribute significantly to free radical scavenging activity, thereby retarding lipid oxidation in the oil medium while imparting a multi-layered olfactory profile dominated by compounds such as anethole, eucalyptol, and linalool [6].

Research on seasoning oils has focused on two main areas: processing optimization and flavor compound analysis. In the realm of process optimization, alternative or refined methods to the conventional oil infusion technique include the frying method, extraction method, combined pressing, and direct addition. The frying method utilizes thermal processing to facilitate flavor extraction and Maillard reactions, although precise temperature control is essential to avoid lipid peroxidation and the development of off flavors [7]. The extraction method is characterized by a relatively brief soaking duration and ease of operation [8,9]. Combined pressing entails the simultaneous pressing of fresh seasonings and oil-bearing crops to extract oil; however, this method may result in elevated acid and peroxide values [10]. The direct addition method involves the incorporation of flavor compounds extracted through microwave-assisted extraction, ultrasonic-assisted extraction, or supercritical CO_2_ extraction. Microwave-assisted extraction is relatively complex; ultrasonic-assisted extraction typically necessitates the use of organic solvents, raising concerns regarding solvent residues. In contrast, supercritical CO_2_ extraction is distinguished by its high safety, absence of solvent residues, and superior flavor retention, albeit at the expense of increased equipment and operational oil [11].

For flavor compound analysis, investigations have centered on single-component seasoning oil systems, such as Sichuan pepper (*Zanthoxy-lum bungeanum*), chili, and scallion oils. Liu et al. used GC-MS to examine volatile flavor compounds in Sichuan pepper oil from various regions, identifying limonene and myrcene as predominant compounds. variations in their concentrations affected the sensory quality [12]. Wang et al. explored flavor changes at different temperatures and identified (E,E)-2,4-decadienal, (E)-2-heptenal, and hexanal as key markers in scallion seasoning oil [13]. Zhang et al. reported that 140–160 °C enriches chili oil flavor, whereas temperatures exceeding 180 °C induce lipid peroxidation and undesirable notes [14]. High-temperature conditions promote the Maillard reaction to generate nitrogen-containing volatiles like alkylpyrazines, with Chun et al. finding glutamine-based systems to be particularly efficient [15]. Further research by Zhang et al. showed that aromatic compounds such as methyl vanillin and styrene in chili oil decreased with prolonged frying, while aldehydes, including hexanal and heptanal, increased over time [16]. Li et al. observed that increasing the raw material-to-oil ratio initially enhanced, then diminished, the aroma and taste of Zanthoxylum Seasoning Oil [11].

Despite advancements in flavor research on seasoning oils, studies have mainly focused on individual spices or specific processing conditions. Research lacks a comprehensive analysis of multi-ingredient oils, particularly regarding synergistic interactions among spices at different temperatures and their influence on flavor compound release. Few studies have analyzed how processing parameters affect the release and transformation of flavor compounds in complex systems. The objectives were to establish the frying temperature via sensory and E-nose analysis and subsequently determine the frying time and the amount of ten representative spices. By analyzing flavor evolution at different temperatures through GC-IMS and GC-MS, this work clarifies the synergistic mechanisms of spices in rapeseed oil, offering a basis for regulating seasoning oil quality during thermal processing.

## 2. Materials and Methods

### 2.1. Optimization of Seasoning Oil

**Figure 1:**
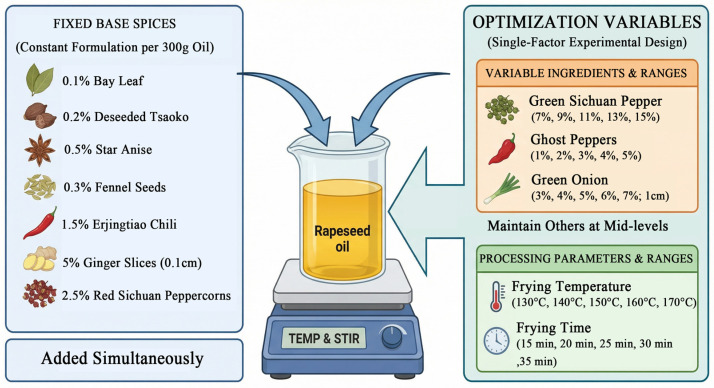
Experimental workflow for the preparation and optimization of seasoning oil.

In this study, flavored rapeseed oil (refined oil) was prepared using 300 g of base oil per trial (Caoyuanliangyou Co., Ltd., Xinjiang, China), with all spices sourced from local markets (Urumqi, Xinjiang, China). Based on preliminary experiments, a standardized base spice formulation was established relative to the oil mass, comprising 0.1% bay leaf, 0.2% deseeded tsaoko fruit, 0.5% star anise, 0.3% fennel seeds, 1.5% Erjingtiao dried chili peppers (coarsely ground powder, unsieved), 5% ginger (0.1 cm slices), and 2.5% red Sichuan peppercorns. To optimize the preparation, a single-factor experimental design was implemented to evaluate five variables across five levels each, centered around the values determined in the preliminary experiments: frying temperature (130, 140, 150, 160, and 170 °C) (stay below the 180 °C smoke point to avoid toxins and save flavor) [17], frying time (15, 20, 25, 30, and 35 min), and the proportions of green Sichuan peppercorns (7, 9, 11, 13, and 15%), ghost peppers (coarsely ground powder, unsieved; 1, 2, 3, 4, and 5%), and green onion (3, 4, 5, 6, and 7%; 1 cm pieces) (Figure 1). During each trial, the oil was heated to the target temperature using a constant-temperature magnetic stirrer, after which the spices were added, and the mixture was maintained at that temperature under continuous stirring for the designated duration. Upon completion, the flavored oil was filtered through gauze to remove spice residues, cooled to room temperature, and stored in sealed containers.

### 2.2. Determination of Physicochemical Indexes

The acid value (AV) and peroxide value (POV) were determined using standard titration methods in accordance with the National Food Safety Standards GB 5009.229-2025 [18] and GB 5009.227-2023 [19], respectively.

### 2.3. Sensory Evaluation

Sensory evaluation was performed by a panel of ten trained assessors (5 males and 5 females, aged 18–32) following the guidelines of GB/T 16291.1-2012 and established protocols [11,20]. The testing took place in individual booths equipped with controlled lighting and ventilation systems. Samples were served at room temperature in odorless, transparent glass containers labeled with random 3-digit codes, using a monadic sequential presentation order. To minimize flavor carry-over effects, panelists cleansed their palates between samples by consuming commercially available plain crackers (IHLAS, Xinjiang, China) and rinsing with warm water (40 °C ± 0.5 °C), followed by a 5-minute rest interval [21]. Each panelist evaluated all samples in triplicate across separate sessions, assessing attributes including taste, aroma, color, and texture (Table S1).

### 2.4. Electronic Nose (E-Nose) Analysis

The analysis was conducted utilizing a C-PEN 3 E-nose (INNOVATE, Germany) equipped with 10 sensors, and the data were processed using WinMuster software (version 1.6.2.18), following the methodology outlined by Zhang et al., with minor modifications [22]. The instrument was powered on and preheated for one hour, after which it was purged with pure air for 30 min to reset the response signal to zero. A sample of seasoning oil (7 g) was placed in a 40 mL headspace vial and equilibrated at 55 °C for 10 min prior to the injection of 600 μL of the sample. The sampling and pre-injection durations were set to 120 and 5 seconds, respectively.

### 2.5. Analysis of Volatile Organic Compounds

The method of Battur et al. [23] was followed in this analysis, where a 1.0 mL sample was placed in a 20 mL headspace vial, with 20 μL of 2-methyl-3-heptanone (10 ppm) as the internal standard. The sample was incubated at 80 °C for 20 min before 300 μL was extracted. Volatile compounds were detected using a FlavourSpec GC-IMS instrument (G.A.S., Germany) with a WAX column measuring 15 × 0.53 mm. The column was maintained at 60 °C, while the IMS was set to 45 °C. High-purity nitrogen served as carrier and drift gases, with analysis lasting 40 min. The headspace sampler was set with a needle temperature of 85 °C, rotation speed of 500 rpm, and incubation of 20 min at 80 °C. Data processing of the volatile organic compounds (VOCs) was performed using LAV software (version 2.2.1, G.A.S., Dortmund, Germany), utilizing the Reporter, Gallery Plot. Compound identification utilized the GC-IMS Library Search module, integrating NIST and IMS databases.

### 2.6. Flavor Profile Analysis

Headspace solid-phase microextraction (HS-SPME) was used to isolate volatile flavor compounds from seasoning oil samples, following a modified protocol based on Li et al. [24]. A 2.0 g sample mass was selected through preliminary validation, ensuring sufficient volatile release while maintaining optimal headspace volume and salting-out conditions, minimizing matrix effects, and enhancing extraction efficiency for GC-MS-based volatile metabolomics analysis. A 2.0 g sample was placed into a headspace vial with a magnetic stir bar and saturated NaCl solution, sealed, and equilibrated at 60 °C with agitation for 5 min. Volatile compounds were extracted using an SPME Arrow (120 μm DVB/CAR/PDMS) fiber, offering ten times greater sensitivity than traditional SPME fibers. The fiber was preconditioned at 250 °C for 5 min before each use, and for 2 hours before initial use. After equilibration, headspace extraction proceeded for 15 min, followed by thermal desorption in the GC injection port at 250 °C for 5 min in splitless mode. GC–MS analysis used a TG-WAXA capillary column (60 m × 0.25 mm × 0.25 μm) with helium carrier gas at a 1.2 mL/min flow rate. The oven temperature program was 40 °C held for 1 min, increased at 3 °C/min to 180 °C, then at 20 °C/min to 230 °C, with a final 10 min hold. The mass spectrometer interface temperature was 230 °C, with electron impact ionization at 70 eV and ion source temperature of 260 °C, operating in full scan mode (*m*/*z* 30–350). Compounds were identified by comparison with the NIST 14.0 mass spectral database, and quantified using peak area normalization to determine percentage composition.

Relative odor activity value (ROAV) was used to characterize the sensory contribution of volatile compounds. In this study, ROAVs were calculated using the metabolites’ relative content data derived from normalized GC-MS peak areas. Compounds with ROAV ≥ 1 were identified as key aroma contributors [25]. ROAVs were calculated according to the methods by Wu et al. [26].

### 2.7. Statistical Analysis

Statistical analyses were performed using SPSS version 28.0 (IBM Corp., Armonk, NY, USA). Data were examined for normality and homogeneity of variance prior to one-way analysis of variance (ANOVA). When the assumptions were met, ANOVA followed by least significant difference (LSD) multiple comparison tests was performed. For multivariate statistical analysis, principal component analysis (PCA) and orthogonal partial least squares discriminant analysis (OPLS-DA) were performed using R software (version 4.3.3, R Core Team). The OPLS-DA model was built and validated using the ‘ropls’ package. The variable importance in projection (VIP) values were calculated to identify the key variables contributing to the group separation, with a threshold of VIP > 1.0. Statistical significance was set at *p* < 0.05. Results are expressed as mean ± standard deviation (SD). Each experiment was conducted in triplicate, and the mean values were used for statistical analysis.

## 3. Results and Discussion

### 3.1. Optimal Experimental Conditions for Seasoning Oil Elaboration

According to the Green Food standard (NY/T 2111-2021) [27], the safety limits for seasoning oil require a peroxide value (POV) below 0.25 g/100 g and an acid value (AV) below 3.0 mg/g. During the optimization process, monitoring results showed that the POV ranged from 0.0444 ± 0.008 to 0.0679 ± 0.002 g/100 g, and the AV ranged from 0.5834 ± 0.006 to 0.6826 ± 0.013 mg/g. These values remained far below the safety thresholds, confirming that the optimization could be safely conducted within the chosen experimental range.

#### 3.1.1. Determination of Optimum Frying Temperature

Seasoning oil must provide a pleasant flavor, appropriate color, and texture. The temperature at which frying is conducted influences taste and aroma [28]. As shown in Figure S1a,b, among the five temperatures tested (130–170 °C for 25 min), samples heated to 160 °C achieved the highest overall taste (27.1) and aroma (23.4) scores (*p* < 0.05). These values represented a 5–15% increase compared to other temperatures, reflecting improved flavor profiles. Conversely, temperature had no significant effect on color or texture attributes (*p* > 0.05), indicating their relative stability within the tested range.

PCA (Figure S1c) accounted for 99.33% of the variance, revealing a temperature-driven shift along PC1. High-impact sensors W3S, W6S, W2S, W2W, and W1W exhibited long vectors along the positive PC1 axis. W5C (aromaaliph) primarily differentiated 160 °C samples along PC2. The radar chart of e-nose response values (Figure S1d) shows that as frying temperature increases, response values for W2W, W1W, W1S, and W2S trend upward. The response value of W1C decreased with rising temperature, reaching its lowest at 170 °C. Compared to 170 °C, W2W and W1W maintained high response values at 160 °C. Analysis of the e-nose responses revealed that only W2W, W1W, W1S, and W2S increased with temperature, identifying sulfur-organics, terpenes, and alcohols as primary drivers of flavor evolution. Although these responses peaked at 170 °C, the simultaneous decline in the W1C (aromatics) signal suggests that excessive heat may trigger the degradation or volatilization of specific aromatic components. In contrast, 160 °C maintained robust responses for key sensors without the loss of W1C integrity observed at higher temperatures, indicating an optimal balance for flavor development. According to Zhang et al., sensors W5S, W6S, W1W, and W2W were most correlated with soybean oil at 165 °C. Based on the sensor characteristics, sulfur-containing compounds contribute significantly to the flavor profile of green onion deep-fried samples. [22]. Considering both sensory analysis and e-nose analysis, 160 °C was selected as the optimal frying temperature.

#### 3.1.2. Determination of Optimum Frying Time

Seasoning oils fried for 15, 20, 25, 30, and 35 min were evaluated for sensory attributes. As shown in Figure S2a,b, the overall sensory score and taste score at 15 min were highest, with taste reaching 27.5, approximately 10–11% higher than at 20 min and 25 min (*p* < 0.05), indicating a more favorable flavor at shorter frying time. Aroma, color, and texture varied within a narrow range across frying durations, with differences below 7% and no significant changes (*p* > 0.05), suggesting that these attributes were relatively insensitive to frying time within the tested range.

E-nose analysis (Figure S2c) effectively discriminated against flavor evolution during frying, with PC1 and PC2 explaining 90.46% of the total variance. A distinct time-dependent trajectory was observed: initial samples (15 min) were characterized by sensors W1W, W2W, and W6S, while mid-stage samples (20–25 min) shifted toward the positive PC1 axis, driven by W5C, W1C, and W3C. At 30–35 min, the profiles migrated toward W3S, W1S, and W2S. Despite increased volatile signals at later stages, sensory scores peaked at 15 min, suggesting that excessive accumulation of compounds beyond this point leads to flavor imbalance. Thus, 15 min is the optimal frying duration. E-nose analysis (Figure S2d) revealed a “decrease-then-increase” trend for sensors W2W, W1W, W1S, and W2S from 15 to 35 min, peaking at 30–35 min. Peak sensory scores at 15 min correlated with moderate volatile release, whereas the surge in sensor responses at 30–35 min likely indicated excessive accumulation of sulfur- and nitrogen-containing compounds. This aligns with Xu et al. [29] and Zhang et al. [16], who noted that prolonged frying significantly alters volatile profiles, explaining the reduced sensory quality at later stages. Thus, 15 min is considered optimal for balancing flavor development.

#### 3.1.3. Determination of the Optimal Amount of Green Sichuan Pepper

As shown in Figure S3a,b, the addition level of Green Sichuan pepper significantly influenced sensory quality. The group with 11% addition achieved the highest overall score (92.9), which was approximately 11–19% higher than other levels (7–15%). Specifically, at this level, the taste score (28.4) and aroma score (23.3) were respectively 15–41% and 9–14% higher than those of other groups (*p* < 0.05), indicating enhanced flavor intensity and aromatic balance. In contrast, color (20.1–23.3) and texture (16.9–18.0) varied by less than 8% across addition levels and showed no significant changes (*p* > 0.05). The improvement in sensory quality can be attributed to the characteristics of Green Sichuan peppercorn. As both a spice and traditional Chinese medicinal herb [30], its fruit possesses a distinctive aroma and numbing flavor that masks undesirable odors, enhances fragrance, improves taste, and stimulates appetite [31].

The PCA biplot (Figure S3c) explains 95.14% of the total variance, effectively discriminating samples based on sensor loading vectors. High-addition groups (11% and 15%) clustered on the positive PC1 axis, driven by sensors W1S (broad-methane), W1W (sulfur-organic), and W2S (broad-alcohol), indicating an enrichment of sulfur-containing compounds and alcohols. Conversely, low-addition groups (7%and 9%) were characterized by W1C/W3C (aromatic) and W5C (aroma-aliph) on the negative PC1 side. Furthermore, W5S (broad range) and W6S (hydrogen) contributed to the PC2 separation. These results demonstrate a systematic volatile shift from aromatics toward sulfur and alcohol-based compounds as addition levels increase. As shown in Figure S3d, at green Sichuan peppercorn levels of 7% and 11%, e-nose response values showed an upward trend, whereas at 13% they decreased for several sensors (W1S, W1W, W2S, W2W, and W5S). At 15%, values increased again, indicating a nonlinear change. Between 7% and 11%, sensors W1S, W1W, and W2S reached peak responses, suggesting higher abundances of terpenes (W1W) and aromatic-sulfur-chlorinated compounds (W2W), as well as alcohols, aldehydes, and ketones (W2S) that contribute to green Sichuan peppercorn aroma. At 13%, responses of multiple sensors declined, with decreases of 26.2% for W1S and 30.6% for W2S, possibly due to excessive addition causing interactions between flavor compounds. An additional level of 11% was identified as optimal.

#### 3.1.4. Determination of the Optimal Amount of Green Pepper

Ghost pepper is valued for its distinctive pungency and flavor profile [32]. Figure S4a shows that a 3% addition level yielded the highest overall sensory score, which was significantly higher than those at 1% and 2% addition levels (*p* < 0.05), with improvements of approximately 6–12%. Further increases beyond 3% did not significantly affect the overall sensory score and resulted in only marginal changes (<2%). Figure S4b presents the sensory attributes; at 3%, taste, aroma, and color reached their highest values, increasing by approximately 7–14%, 9–18%, and 21–26%, respectively, compared with lower concentrations (*p* < 0.05), whereas texture varied by less than 7% across all addition levels and showed no meaningful practical change.

The PCA biplot (Figure S4c) explains 98.99% of the total variance, effectively discriminating samples by addition levels. High-addition groups (4%–5%) correlate with sensors W1S, W1W, and W2S on the positive PC1 axis, indicating an enrichment of sulfur-organic compounds and alcohols. Conversely, low-addition groups (1%–2%) align with W1C, W3C, and W5C on the negative PC1 side, reflecting a dominance of aromatic and aliphatic compounds. W3S (methane-aliph) primarily drives the separation along PC2. Analysis of the e-nose response values (Figure S4d) revealed that as the addition level of ghost pepper powder increased from 1% to 5%, the responses of W1W, W2W, W5S, and W1S changed markedly. These changes reflected increases in the relative concentrations of organosulfur compounds, terpenes, aromatic and sulfur-containing compounds, nitrogen oxides, and short-chain alkane volatiles. Notably, at an additional level of 3%, the release of these compounds peaked. Therefore, 3% was identified as the optimal addition level of ghost pepper powder.

#### 3.1.5. Determination of the Optimal Amount of Green Onion

As shown in Figure S5a, a 6% green onion addition level yielded the highest overall sensory score, which was significantly higher than those of the other addition levels (*p* < 0.05), with improvements of approximately 3–7% compared with the 3–5% groups and about 6% compared with the 7% group. Further deviation from the 6% level resulted in reduced or only marginal changes in the overall sensory score. Figure S5b presents the sensory attributes; at 6%, taste reached its highest value, increasing by approximately 2–7% relative to the other concentrations (*p* < 0.05). Aroma and color showed relatively small variations across addition levels (≤5%), despite reaching their maximum values at 6%, whereas texture varied by less than 3% among all groups and showed no meaningful practical change. These results can be attributed to the characteristics of green onions, which possess a strong aroma and are frequently utilized as a seasoning or spice to contribute substantial culinary value. Following thermal extraction, its fresh green aroma diminishes, while savory and roasted notes are accentuated, effectively masking undesirable flavors and enhancing the taste and flavor of dishes [13].

PCA of the E-nose data (Figure S5c) showed that PC1 and PC2 accounted for 95.4% of the total variance. Loading vector analysis revealed that sensors W2W, W1W, W2S, and W6S (sulfur compounds/alcohols) were the primary markers driving the separation of the 6%–7% groups along negative PC1. In contrast, W1C, W3C, and W5C characterized the 3% group. The shorter vectors for W1S and W3S indicated their minimal contribution, confirming that sample discrimination relies on specific high-impact chemical markers. This indicates that these components captured the majority of the flavor variation among the samples. As illustrated in Figure S5d, increasing the green onion addition level from 3% to 7% resulted in a discernible pattern in the E-nose response values. At an addition level of 3–4%, aromatic compounds were predominant, resulting in a relatively simple flavor profile. At 5–6%, there was an increased release of organosulfur compounds and oxygen-containing volatiles, which enriched the flavor profile. When the addition level exceeded 6%, the release of certain sulfur compounds further increased, potentially leading to an excessively intense flavor. Consequently, 6% was identified as the optimal additional level of green onion.

### 3.2. Aromatic Compounds and Flavor Profile

Zhang et al. identified temperature as a critical factor influencing flavor compound formation, particularly during the Maillard reaction [22]. Ni et al. [33] and Sun et al. [34] reported that temperature variations affect the generation of volatile aroma components, with the evaluated temperature ranges being 60–120 °C for fried mountain pepper oil and 110–160 °C (including initial temperatures of 110–125 °C and final temperatures of 145–160 °C) for fried garlic oil. In optimization, we developed a seasoning oil with ten seasonings, resulting in more diverse flavor compounds compared to previous single-seasoning studies. It was essential to examine the temperature’s influence on these flavor compounds. In determining the conditions, we found 160 °C was optimal. We employed GC-IMS and GC-MS to investigate flavor compound changes at various temperatures (150 °C, 160 °C, and 170 °C) to understand why 160 °C is optimal.

#### 3.2.1. Volatile Organic Compounds

**Figure 2:**
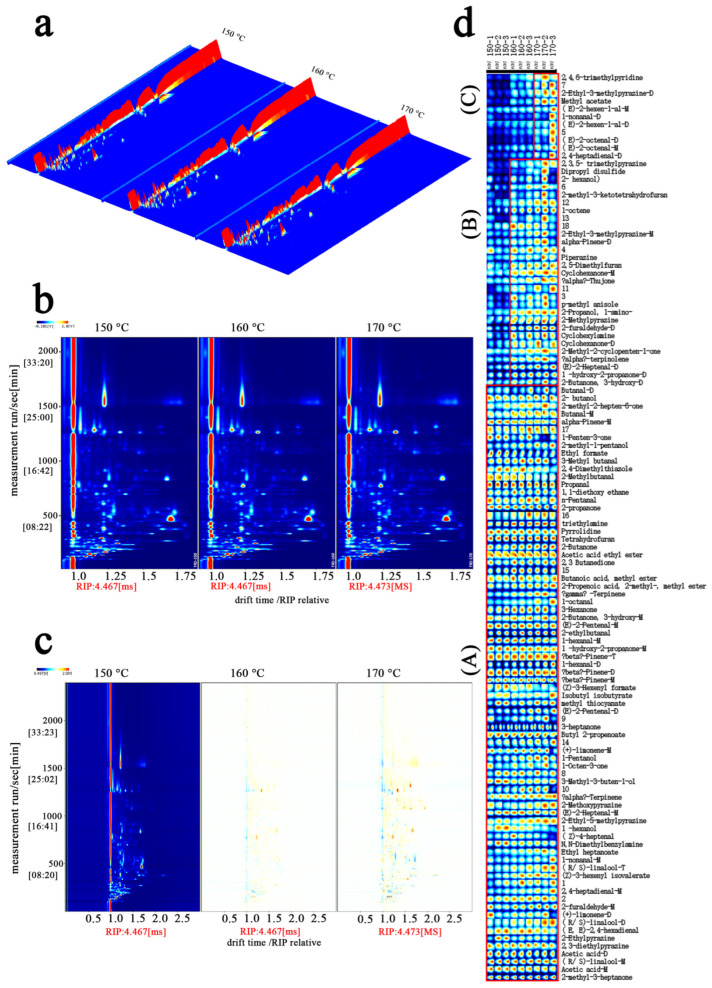
GC-IMS spectra of VOCs in the sample. (**a**) 3D GC-IMS spectrum; (**b**) GC-IMS spectrum (direct comparison); (**c**) GC-IMS spectrum (differential comparison); (**d**) fingerprint database (gallery plot) of VOCs, Part (A) presents the segment with relatively stable volatile compound profiles across temperatures; part (B) represents the segment showing variations between 150 °C and 160 °C; part (C) corresponds to the segment with changes observed at 170 °C.

Figure 2 shows results visualized as 3D topographic maps, difference plots, and Gallery Plot diagrams. The X-axis represents drift time, and the Y-axis represents retention time. The red vertical line on the left corresponds to the reactant ion peak (RIP), and the blue area indicates background. [35,36].

To evaluate VOC changes at different frying temperatures, multiple visualization methods were applied. As shown in Figure 2a, the 3D topographic map shows overall differences among samples. Because subtle variations are difficult to distinguish in the 3D view, a 2D top-view plot was used. As shown in Figure 2b, VOC peak distributions were similar across temperature groups, indicating stable basic composition. However, variations in peak intensity and color suggested that temperature influenced compound abundance. VOCs were distributed within 500–1500 s retention time and 1.0–1.75 ms drift time ranges, with composition patterns similar among the three seasoning oil samples.

For differential analysis, the 150 °C sample served as a reference. Compared with the 160 °C sample (Figure 2c), the difference plot appeared predominantly red with moderate intensity and few blue regions in the low-retention-time area. Comparison with the 170 °C sample showed both red and blue regions, with dark-red areas indicating substantial increases in specific compounds, while blue regions showed marked decreases in certain components. These differences reflect pronounced alterations in flavor-active compounds under high-temperature conditions.

Using the GC-IMS Gallery Plot, a fingerprint profile of volatile flavor compounds in seasoning oil was established (Figure 2d). The analysis identified 74 volatile compounds, including 20 aldehydes, 15 nitrogen-containing compounds, 12 ketones, 11 terpenes, 9 esters, and 7 alcohols. The plot was divided into regions A, B, and C based on the relative concentration shifts across temperatures. At 160 °C (Region B), a significant increase was observed in several key compounds compared to the 150 °C sample: 2-furaldehyde-D (+61.83%), 2-methylpyrazine (+41.74%), (E)-2-heptenal (+38.30%), α-terpinolene (+35.44%), 2,5-dimethylfuran (+34.24%), and γ-terpinene (+29.49%). As the temperature further increased to 170 °C (Region C), the concentrations of 2,4-heptadienal-D, 1-nonanal-D, and methyl acetate continued to rise by 30.12%, 44.19%, and 30.88%, respectively, relative to the 160 °C group. These results indicate that temperature significantly influences the abundance and composition of volatile profiles.

The evolution of VOCs is driven by temperature-dependent chemical pathways, primarily the Maillard reaction and lipid oxidation. The significant rise in 2-methylpyrazine at 160 °C reflects the enhanced interaction between sugar-derived carbonyls and amino groups under moderate heat, contributing nutty and roasted characteristics [37]. Similarly, the accumulation of 2-furaldehyde-D and acetol suggests that these conditions accelerate the thermal degradation and dehydration of pentose sugars and polysaccharides [38]. The increase in γ-terpinene and α-terpinolene is likely associated with the thermal release and rearrangement of monoterpenes from the spice matrices [39]. These combined processes at 160 °C establish a complex flavor profile through concurrent Maillard and lipid oxidation reactions.

The GC-IMS “micro-fingerprint” provides a molecular basis for the E-nose “macro-fingerprint” (Figure S1c,d). The high diversity of aldehydes and ketones at 160 °C (Region B) explains the specific response of sensor W5C, while the shift toward nitrogen compounds and alcohols at 170 °C (Region C) correlates with sensors W1W, W2W, and W2S. However, our findings suggest a critical threshold: while 150 °C provides insufficient energy for robust flavor development, 170 °C promotes excessive oxidation, as evidenced by the sharp rise in 1-nonanal and 2,4-heptadienal. This over-accumulation of oxidation-derived volatiles likely leads to an imbalanced aroma profile. This chemical shift explains why the 160 °C sample received the highest sensory scores, as it achieved the most harmonious balance of flavor-active compounds. Therefore, 160 °C represents the optimal processing temperature for a high-quality volatile profile.

#### 3.2.2. Flavor Profile

##### GC-MS Identification of Volatile Characteristics in Seasoning Oil

**Figure 3:**
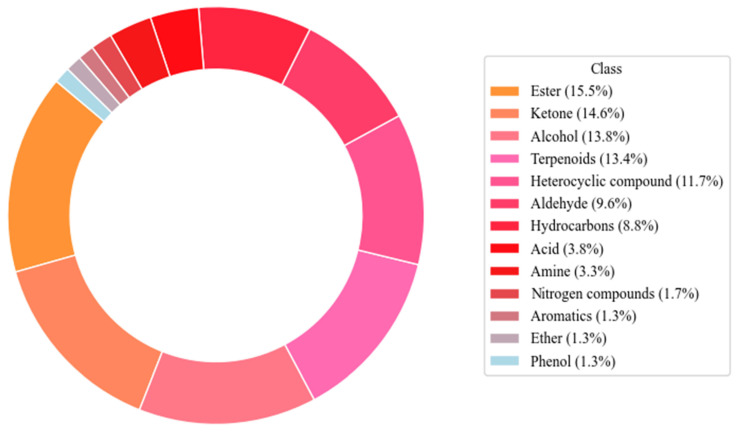
Percentage of volatile compounds, by chemical class, identified in the seasoning oil.

A total of 239 VOCs were identified in the seasoning oil at different frying temperatures (Table S2). Figure 3 shows the distribution of volatile metabolite categories in seasoning oil. Esters form the largest segment at 15.5%, followed by ketones (14.6%), alcohols (13.8%), and terpenoids (13.4%). These four categories comprise 57.3% of the total. Heterocyclic compounds account for 11.7%, aldehydes for 9.6%, and hydrocarbons for 8.8%. Acids represent 3.8%, amines 3.3%, nitrogen compounds 1.7%, and aromatic compounds, ethers, and phenolic compounds each constitute 1.3%.

The dominance of esters, ketones, alcohols, and terpenoids indicates their significant role in the aroma profile. Esters and ketones are linked to fruity or buttery aromas, alcohols contribute fresh and mildly sweet scents, while terpenoids provide herbal and floral characteristics [40]. Prior studies indicate that terpenoid levels in rapeseed oil are generally low and not considered major volatile components [41]. However, our research suggests that terpenoids in seasoning oils from rapeseed oil are significant active components, potentially influencing their physicochemical properties and bioactivity [42]. Under specific processing conditions, terpenoids may concentrate, enhancing their impact on flavor, as they are primary volatile compounds released during frying [43]. Consistent with this, Liu et al. identified terpenoids as key contributors to Zanthoxylum bungeanum fried pepper oil flavor [44]. Compared to the volatile profile of single-source Sichuan pepper oil described by Liu et al., the multi-ingredient seasoning oil examined here exhibits a broader chemical spectrum [12]. This expanded range indicates complex synergistic interactions among spices and auxiliary components, resulting in a more profound, multidimensional flavor profile than single-ingredient formulations.

##### Multivariate Statistical Analysis of VOCs

**Figure 4:**
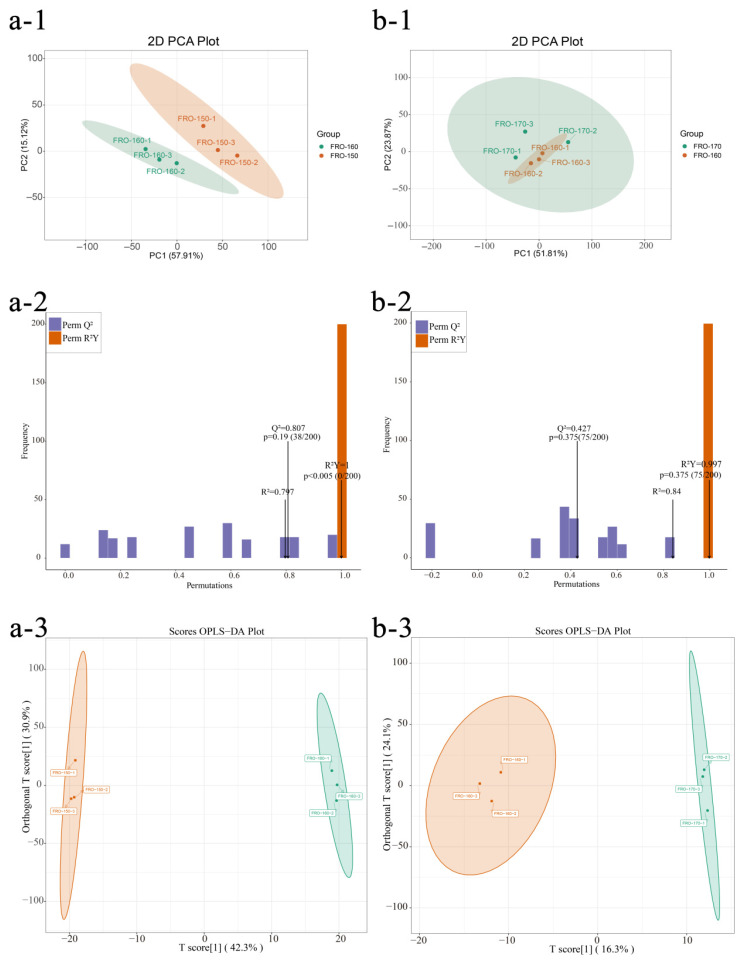
Multivariate statistical analysis of samples at different frying temperatures. Group a (150 °C vs. 160 °C) and Group b (160 °C vs. 170 °C) are analyzed using PCA score plots (**a-1**,**b-1**). Validation plots (**a-2**,**b-2**) are derived from a 200-cycle permutation test to evaluate R^2^X, R^2^Y, and Q^2^ parameters, where R^2^X and R^2^Y represent the explained variance of the X and Y matrices, and Q^2^ reflects the model’s predictive ability. The results show Q^2^ > 0.5 and *p* < 0.05, indicating that the models have strong predictive power and are statistically superior to random-assignment models. Panels (**a-3**,**b-3**) present the OPLS-DA score plots.

To explore variations between adjacent temperatures, pairwise PCA was performed (Figure 4(a-1,b-1)). PC1 and PC2 accounted for 73.03% (150/160 °C) and 75.68% (160/170 °C) of the total variance. This high cumulative variance (>70%) suggests that these primary components capture a substantial proportion of the VOC profile diversity, indicating that the observed sample distribution reflects temperature-induced chemical shifts. These findings illustrate how frying temperature influences the volatile composition of seasoning oils. Orthogonal partial least squares discriminant analysis (OPLS-DA), a supervised multivariate statistical technique [45], was used to identify volatile metabolite changes in seasoning oils at different frying temperatures. The OPLS-DA validation plots for seasoning oils at the three frying temperatures (Figure 4(a-2,b-2)) showed excellent model parameters, with R2X, R2Y, and Q2 as predictive metrics. As shown in Figure 4(a-3,b-3), the model demonstrated validity and strong predictive power, making it suitable for investigating differences among seasoning oils at various frying temperatures.

##### Impact of Frying Temperature on VOCs

**Figure 5:**
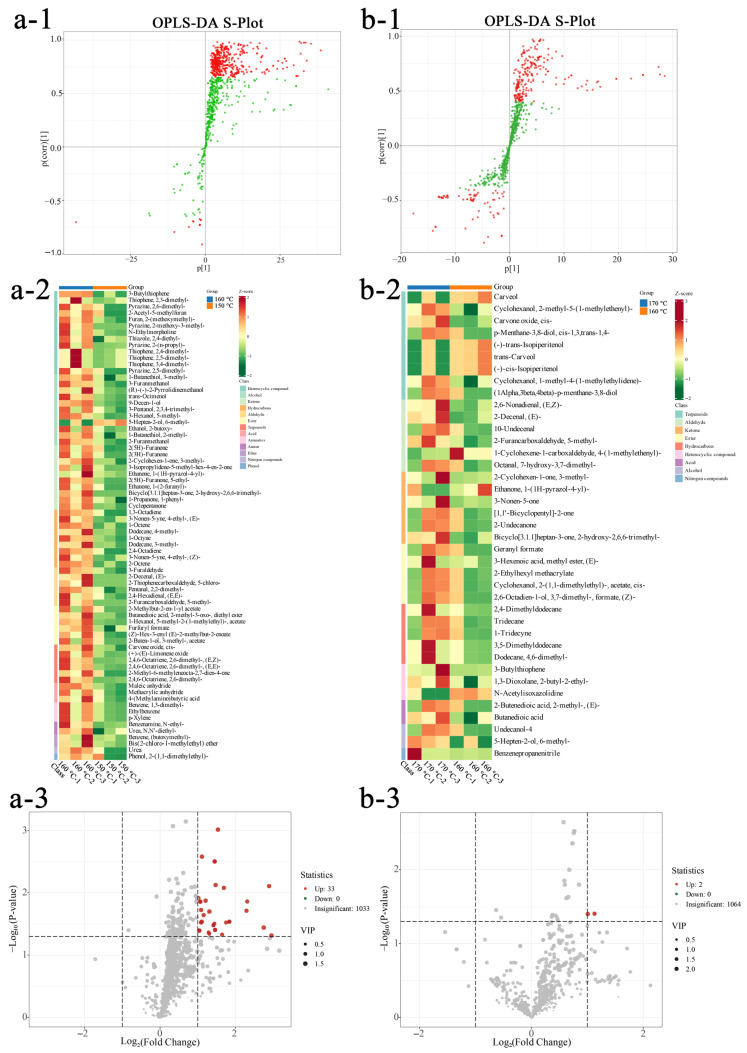
Pairwise comparative analysis of volatile organic compounds in samples from different temperature groups. Group a is 150 °C vs. 160 °C, and Group b is 160 °C vs. 170 °C. (**a-1**,**b-1**) red dots represent variables with strong contributions at the plot extremes, while green dots represent variables with weak contributions near the center. OPLS-DA S-plots; (**a-2**,**b-2**) are cluster heatmaps of differential metabolites; and (**a-3**,**b-3**) are volcano plots of differential metabolites.

Key differential VOCs were identified using a dual-filtering strategy. The synergistic screening based on VIP (>1), *p*-value (<0.05), and |log2FC| (>1) provides a reliable framework for identifying temperature-induced transformations. This approach ensures markers are both discriminative and statistically robust, providing a solid basis for subsequent chemical interpretation. [46]. As illustrated in Figure 5(a-1,4b-1), 73 VOCs with VIP > 1 were identified between 150 °C and 160 °C, whereas 39 VOCs with VIP > 1 were identified between 160 °C and 170 °C. Differential analysis of VOCs across temperature groups (log2FC > 1, *p* < 0.05) further indicated that 33 VOCs were upregulated at 160 °C relative to 150 °C (Figure 5(a-3)). As depicted in Figure 5(a-2), the magnitude of change increased with frying temperature, most notably for terpenes, alcohols, and esters. Representative increases included 3-butylthiophene (0.015–0.159 μg/g), 2-(methoxymethyl)furan (0.176–0.986 μg/g), 5-ethyl-2(5H)-furanone (0.031–0.278 μg/g), 2-methoxy-3-methylpyrazine (0.049–0.535 μg/g), and 2-hydroxy-2,6,6-trimethylbicyclo [3.1.1]heptan-3-one (0.005–0.064 μg/g). In contrast, only two VOCs were upregulated at 170 °C relative to 160 °C (Figure 5(b-3)). As shown in Figure 5(b-2), 5-methyl-2-furancarboxaldehyde (an aldehyde) and cis-carvone oxide exhibited pronounced temperature-dependent fluctuations.

The marked increase in terpenes, alcohols, and esters at 160 °C identifies this temperature as a pivotal threshold for flavor evolution, which is consistent with previous observations regarding temperature-dependent volatile formation [47]. The identified heterocycles and ketones likely originated from Maillard reactions and lipid oxidation. These compounds contribute significantly to the sensory profile: pyrazines and thiophenes provide roasted and nutty notes, furans and furanones offer sweet or fruity characters, and bicyclic ketones contribute woody or herbal notes [48]. The minimal increase in VOCs at 170 °C suggests a plateau or a shift toward thermal degradation and advanced Maillard-type reactions at higher temperatures [49].

The correlation analysis of VOCs at 150 °C, 160 °C, and 170 °C revealed pronounced temperature-dependent release patterns and key inflection points. At 160 °C compared with 150 °C, most compounds exhibited strong and significant positive correlations; for example, bicyclo [3.1.1]heptan-3-one and its 2-hydroxy-2,6,6-trimethyl derivative showed r = 0.994 (*p* < 0.05). Many furan compounds displayed strong correlations with terpenes (r > 0.9), while alcohols and aldehydes generally showed moderate correlations (0.8 < r < 0.9). This pervasive pattern of positive correlations indicates that, within this temperature range, the release of various compounds occurs in a relatively coordinated manner.

**Figure 6:**
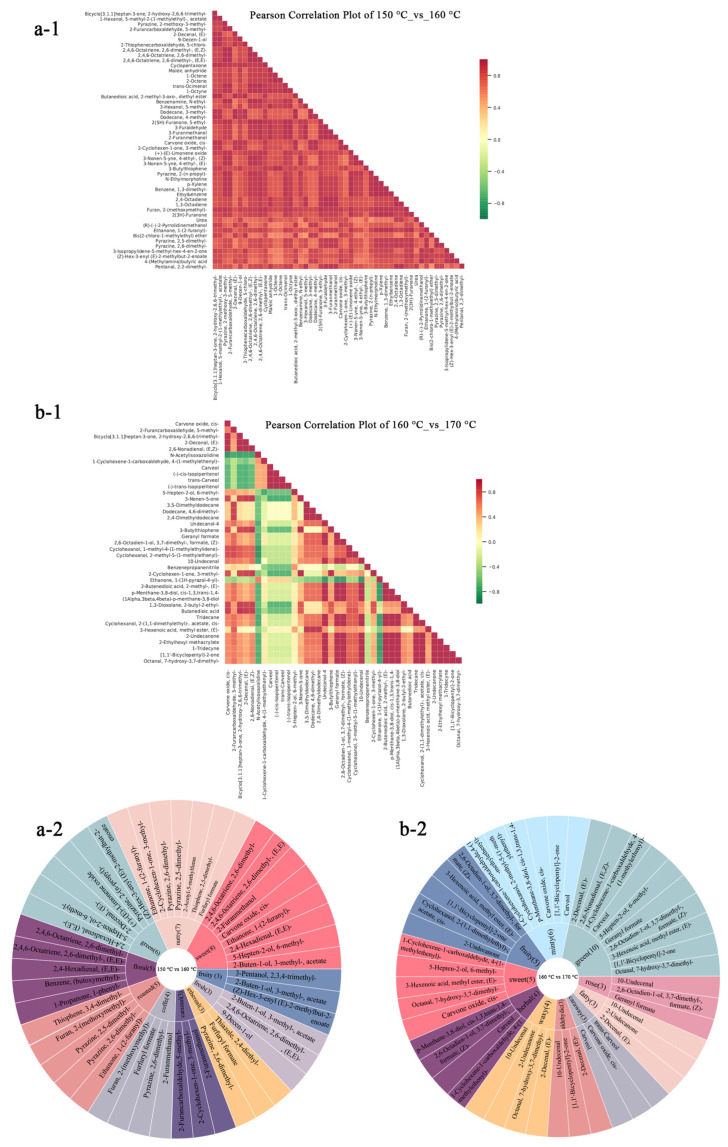
Correlation heatmap and flavor wheel of differential metabolites between samples at different temperatures. Figures (**a-1**,**b-1**) show the flavor wheels of the differential metabolites; (**a-2**,**b-2**) display the correlation heatmaps of the differential metabolites. Group a represents 150 °C vs. 160 °C, and Group b represents 160 °C vs. 170 °C.

However, as shown in Figure 6(b-1), upon increasing the temperature to 160 °C and 170 °C, the correlation pattern changed: ethanone, 1-(1H-pyrazol-4-yl)- exhibited significant negative correlations with various oxygen-containing alcohols and carboxylic acids, particularly undecanol-4, geranyl formate, 2-butenedioic acid, and p-menthane-3,8-diol. Similarly, N-acetylisoxazolidine showed significant negative correlations with cis-carvone oxide and related cyclic alcohols. In contrast, carveol and its three isomers [including (-)-cis-isopiperitenol, trans-carveol, and (-)-trans-isopiperitenol] formed a cluster of highly positively correlated compounds, while this isomer group generally exhibited negative correlations with oxidized ketones such as carvone oxide.

These observed shifts in correlation suggest potential interactions among different classes of compounds at 160 °C and 170 °C. These changes might be linked to variations in formation, degradation, or transformation pathways—such as lipid oxidation or Maillard reaction stages—although the current correlation data are insufficient to confirm specific causal mechanisms. In summary, the VOC profiles suggest that 160 °C is characterized by a distinct and highly integrated correlation pattern. This may indicate a more balanced chemical development at this temperature, providing a potential chemical basis for the harmonious aroma observed in sensory evaluations.

Volatile compounds are critical determinants of food flavor [50]. This study identified the top ten differential metabolites with the highest frequency of flavor annotation in each comparison group, as illustrated in Figure 6(a-2,b-2). The sensory and volatile profiles at 160 °C highlight its optimality for thermal processing, markedly enhancing desirable attributes over 150 °C such as sweetness (eight types, via aldehydes like 2,4-octadienal, hexanoic acid, and furans like 1-(2-furanyl)-ethanone), nutty flavor (7 types, from pyrazines like 2,5-dimethylpyrazine, furans, and thiophenes), green notes (6 types, from unsaturated aldehydes like 2-decenal, esters, and trienes), floral (5 types, with aromatics and alcohols), roasted/coffee/caramel (12 types total, via Mail-lard/sulfur reactions), and others through balanced Maillard reactions and caramelization, yielding a complex, harmonious aroma [33]. In contrast to 170 °C, the 160 °C group appeared to retain more green, fruity, and sweet notes while exhibiting fewer waxy off-flavors (such as octanal), potentially reducing the risk of over-processing. The combined chemical and sensory evidence indicate that 160 °C may have promoted the accumulation of desirable aroma-active compounds while limiting unfavorable volatiles, thus supporting a more complex and fresh profile. Consequently, this temperature was found to balance sweetness, nutty richness, and roasted depth more effectively, which corresponded with the highest sensory evaluations.

##### ROAVs Analysis

ROAV was employed to evaluate the contribution of individual VOCs to the overall aroma, with compounds exceeding a value of 1 considered key flavor contributors [51]. As shown in Table 1, among these, 2-methoxy-3-(1-methylethyl)-pyrazine was found to have the highest ROAV (100) across all groups, suggesting its role as a major contributor to the characteristic nutty, beany, and roasted notes. As the temperature rose from 150 °C to 170 °C, the ROAV of dihydro-2-methyl-3(2H)-furanone significantly decreased from 92.12 ± 3.94 to 67.60 ± 11.73, which may be associated with the perceived reduction in sweet and caramel-like attributes. Simultaneously, the ROAV of β-damascone reached its peak at 160 °C (8.83 ± 0.41); this could potentially be attributed to the thermal liberation of terpenoids from carotenoid degradation, although further mechanistic studies are needed. While lipid-derived aldehydes remained relatively stable, the increase in ethyl 3-cyclohexenecarboxylate at 170 °C might have contributed to greater ester-like complexity under high-heat conditions [52]. In view of these findings, the flavor profile appeared to shift from “sweet-dominant” at 150 °C to “harmonious and complex” at 160 °C, before moving toward a “heavily roasted” character at 170 °C, suggesting 160 °C as a favorable temperature for balanced flavor development.

## 4. Conclusions

This study determined the optimal preparation parameters for a multi-ingredient seasoning oil and elucidated the impact of thermal processing on flavor release. The results established that frying at 160 °C for 15 min using a precise formulation—11% green Sichuan pepper, 6% green onion, 5% ginger, 3% ghost pepper, 2.5% red Sichuan pepper, 1.5% dried Erjingtiao chili, 0.5% star anise, 0.3% fennel seeds, 0.2% deseeded tsaoko, and 0.1% bay leaf—yielded the superior sensory profile. GC–IMS analysis revealed that processing at 160 °C significantly enriched key volatile markers, specifically increasing the contents of 2-methylpyrazine, 2-furaldehyde-D, γ-terpinene, triethylamine, α-terpinolene, 2,5-dimethylfuran, cyclohexylamine, and (E)-2-heptenal-D compared to 150 °C and 170 °C. Furthermore, GC–MS analysis confirmed that 160 °C acted as a critical thermodynamic threshold, upregulating 33 volatile compounds (e.g., 2-methoxy-3-methylpyrazine) while minimizing degradation. ROAV analysis identified 2-methoxy-3-(1-methylethyl)-pyrazine as the most potent aroma-active compound, defining the oil’s characteristic nutty notes. These findings provide scientifically grounded parameters for industrial seasoning oil production and offer molecular insights into optimizing the “generation-degradation” balance of flavor compounds during thermal processing.

## Figures and Tables

**Table 1:** Volatile compounds (ROAV ≥ 1) in samples fried at 150 °C, 160 °C, and 170 °C.

Number	Compounds	Class I	Formula	Odor	Threshold	ROAV		
						150 °C	160 °C	170 °C
1	2,4-Undecadienal	Aldehyde	C_11_H_18_O	green, buttery, spicy,	0.00001	0.94 ± 0.06	1.02 ± 0.05	0.99 ± 0.14
2	Ethyl 3-cyclohexenecarboxylate	Ester	C_9_H_14_O_2_	ND	0.000003	2.53 ± 0.40	2.39 ± 0.06	3.34 ± 0.24
3	5-Nonenal, (E)-	Aldehyde	C9H16O	ND	0.007	1.32 ± 0.02	1.33 ± 0.01	1.31 ± 0.01
4	dihydro-2-methyl-3(2H)-furanone	Ketone	C_5_H_8_O_2_	sweet, solvent,	0.000005	92.12 ± 3.94	71.18 ± 6.80	67.60 ± 11.73
5	3-Mercapto-3-methylbutyl formate (ester)	Ester	C_6_H_12_O_2_S	sulfury, caramel, onion, roasted coffee,	0.000002	0.96 ± 0.03	1.13 ± 0.11	0.99 ± 0.25
6	1-Nonen-3-one	Ketone	C_9_H_16_O	pungent, mushroom	0.000001	36.53 ± 0.42	34.82 ± 0.54	32.96 ± 2.15
7	Benzoic acid, methyl ester	Ester	C_8_H_8_O_2_	phenol, wintergreen	0.00052	1.25 ± 0.02	1.27 ± 0.01	1.25 ± 0.01
8	Pyrazine, 2-methoxy-3-(1-methylethyl)-	Heterocyclic compound	C_8_H_12_N_2_O	beany, pea, earthy, chocolate,	0.000002	100.00 ± 0.00	100.00 ± 0.00	100.00 ± 0.00
9	(Z,Z)-3,6-Nonadienal	Aldehyde	C_9_H_14_O	fatty, cucumber	0.00005	2.72 ± 0.05	2.76 ± 0.01	2.71 ± 0.02
10	Non-8-enal	Aldehyde	C_9_H_16_O	smoky, plastic	0.0002	6.54 ± 0.10	6.59 ± 0.04	6.50 ± 0.05
11	β-damascone	Terpenoids	C_13_H_20_O	fruity, floral, berry,	0.000002	7.97 ± 0.50	8.83 ± 0.41	8.46 ± 1.26
12	Pyrazine, 2-methoxy-3-(2-methylpropyl)-	Heterocyclic compound	C_9_H_14_N_2_O	green bell pepper, pea	0.000002	5.79 ± 0.68	5.77 ± 0.39	5.40 ± 0.81
13	2(5H)-Furanone, 5-ethyl-3-hydroxy-4-methyl-	Ketone	C_7_H_10_O_3_	sweet, fruity, caramel,	0.000002	1.90 ± 0.25	1.94 ± 0.29	1.98 ± 0.27
14	6-Nonenal, (E)-	Aldehyde	C_9_H_16_O	ND	0.000022	2.41 ± 0.03	2.43 ± 0.01	2.39 ± 0.02
15	Phenol, 2-chloro-4-methyl-	Phenol	C_7_H_7_ClO	ND	0.0003	3.31 ± 0.05	3.36 ± 0.03	3.31 ± 0.03
16	3-Octen-2-one	Ketone	C_8_H_14_O	earthy, spicy	0.00003	4.16 ± 0.90	3.88 ± 0.05	3.04 ± 0.96

ND: Not detected. Odor: the scent descriptions are sourced from http://www.thegoodscentscompany.com, http://perflavory.com/, http://www.odour.org.uk/odour/index.html, or http://foodflavorlab.cn/#/home, The above websites were all accessed on 15 December 2025.

## Data Availability

The original contributions presented in this study are included in the article. Further inquiries can be directed to the corresponding author.

## Supplementary Materials

The following supporting information can be downloaded at https://www.mdpi.com/article/10.3390/foods15040626/s1. Figure S1: Effects of frying temperature on sensory evaluation and E-nose responses of seasoning oil. (a) Overall sensory score; (b) individual sensory attribute scores; (c) e-nose principal component analysis (PCA); (d) e-nose radar plot. Different letters indicate significant differences (*p* < 0.05). Note: W1C, aromatics; W5S, broad-range; W3C, aromatics; W6S, hydrogen; W5C, aromatic/aliphatic hydrocarbons; W1S, broad-methane; W1W, organic sulfur; W2S, alcohols/aldehydes/ketones; W2W, sulfur- and chlorinated compounds; W3S, methane/aliphatic hydrocarbons; Figure S2: Effects of frying time on sensory evaluation and E-nose responses of seasoning oil. (a) overall sensory score; (b) sensory attribute scores; (c) e-nose principal component analysis (PCA); (d) e-nose radar plot. Different letters indicate significant differences (*p* < 0.05). Note: W1C, aromatics; W5S, broad-range; W3C, aromatics; W6S, hydrogen; W5C, aromatic/aliphatic hydrocarbons; W1S, broad-methane; W1W, organic sulfur; W2S, alcohols/aldehydes/ketones; W2W, sulfur-and chlorinated compounds; W3S, methane/aliphatic hydrocarbons. Figure S3: Effects of green Sichuan pepper addition level on sensory evaluation and E-nose responses of seasoning oil. (a) overall sensory score; (b) individual sensory attribute scores; (c) e-nose principal component analysis (PCA); (d) e-nose radar plot. Different letters indicate significant differences (*p* < 0.05). Note: W1C, aromatics; W5S, broad-range; W3C, aromatics; W6S, hydrogen; W5C, aromatic/aliphatic hydrocarbons; W1S, broad-methane; W1W, organic sulfur; W2S, alcohols/aldehydes/ketones; W2W, sulfur-and chlorinated compounds; W3S, methane/aliphatic hydrocarbons. Figure S4: Effects of ghost pepper addition level on sensory evaluation and E-nose responses of seasoning oil. (a) overall sensory score; (b) individual sensory attribute scores; (c) e-nose principal component analysis (PCA); (d) e-nose radar plot. Different letters indicate significant differences (*p* < 0.05). Note: W1C, aromatics; W5S, broad-range; W3C, aromatics; W6S, hydrogen; W5C, aromatic/aliphatic hydrocarbons; W1S, broad-methane; W1W, organic sulfur; W2S, alcohols/aldehydes/ketones; W2W, sulfur- and chlorinated compounds; W3S, methane/aliphatic hydrocarbons. Figure S5. Effects of green onion addition level on sensory evaluation and E-nose responses of seasoning oil. (a) overall sensory score; (b) individual sensory attribute scores; (c) e-nose principal component analysis (PCA); (d) e-nose radar plot. Different letters indicate significant differences (*p* < 0.05). Note: W1C, aromatics; W5S, broad-range; W3C, aromatics; W6S, hydrogen; W5C, aromatic/aliphatic hydrocarbons; W1S, broad-methane; W1W, organic sulfur; W2S, alcohols/aldehydes/ketones; W2W, sulfur- and chlorinated compounds; W3S, methane/aliphatic hydrocarbons. Table S1: Sensory evaluation score for seasoning oil. Table S2: Identified volatile components in seasoning oils using gas chromatography–mass spectrometry (GC-MS).
