# Supplementary material for: Effects of Frying Temperature and Composite Spices on the Release Characteristics of Rapeseed Seasoning Oil
Source: Foods. 2026 Feb 9;15(4):626. doi: 10.3390/foods15040626 (PMC12939693; doi:10.3390/foods15040626)
Supplement: Supplementary file 1 [file foods-15-00626-s001.zip › foods-4114247-supplementary.pdf]

Table S1. Sensory evaluation score for seasoning oil

Indicator	Sensory Description	Score
Taste	The aroma is rich, with the refreshing numbing fragrance and spicy aroma of Sichuan peppercorn standing out. It has a lasting aftertaste with no off-flavors.	28~30
	The numbing and spicy flavor is too strong, but the texture is appropriate. The aftertaste is a bit short, with no off-flavors.	25~27
	The numbing flavor is average, the spiciness is average, the texture is average, the aftertaste is short, and there is a slight off-flavor.	22~24
	Overall aroma is average, with notes of Sichuan pepper and chili. The scent is moderately balanced, with no off-odors.	0~21
Aroma	The overall aroma is rich, with a prominent peppery and numbing fragrance. The chili aroma is pleasant, well-balanced, and there are no off-odors.	23~25
	The overall aroma is average, with a peppery and spicy fragrance. The scent is moderately balanced, with no off-odors.	20~22
	The overall aroma is not pronounced, with a numbing pepper fragrance and a slightly pungent chili scent. The scents are not well-balanced, and there is a slight off-odor.	17~19
	Overall, there is no fragrance, no peppery aroma, the chili is pungent, the scent lacks harmony, and there is a noticeable off-odor.	0~16
Color	The oil has a clear, transparent appearance, is bright red and glossy, with no abnormal coloration.	23~25
	The oil is clear and transparent in color, with a slightly light or slightly deep hue, glossy, and free from abnormal colors.	20~22
	The oil appears slightly cloudy, with colors that are not well-coordinated. It has a slight sheen and shows some discoloration.	17~19
	The oil appears cloudy and dull in color, lacking luster, with an abnormal hue.	0~16
Texture	The oil is fluid in form, moderately viscous, and free of impurities.	18~20
	The oil appears as a liquid, slightly viscous or slightly thin, with slight impurities.	15~17
	The oil has a fluid form, which can be relatively thick or thin, and contains a considerable amount of impurities.	12~14
	The oil is in a fluid state, either very thick or very thin, with many impurities.	0~11

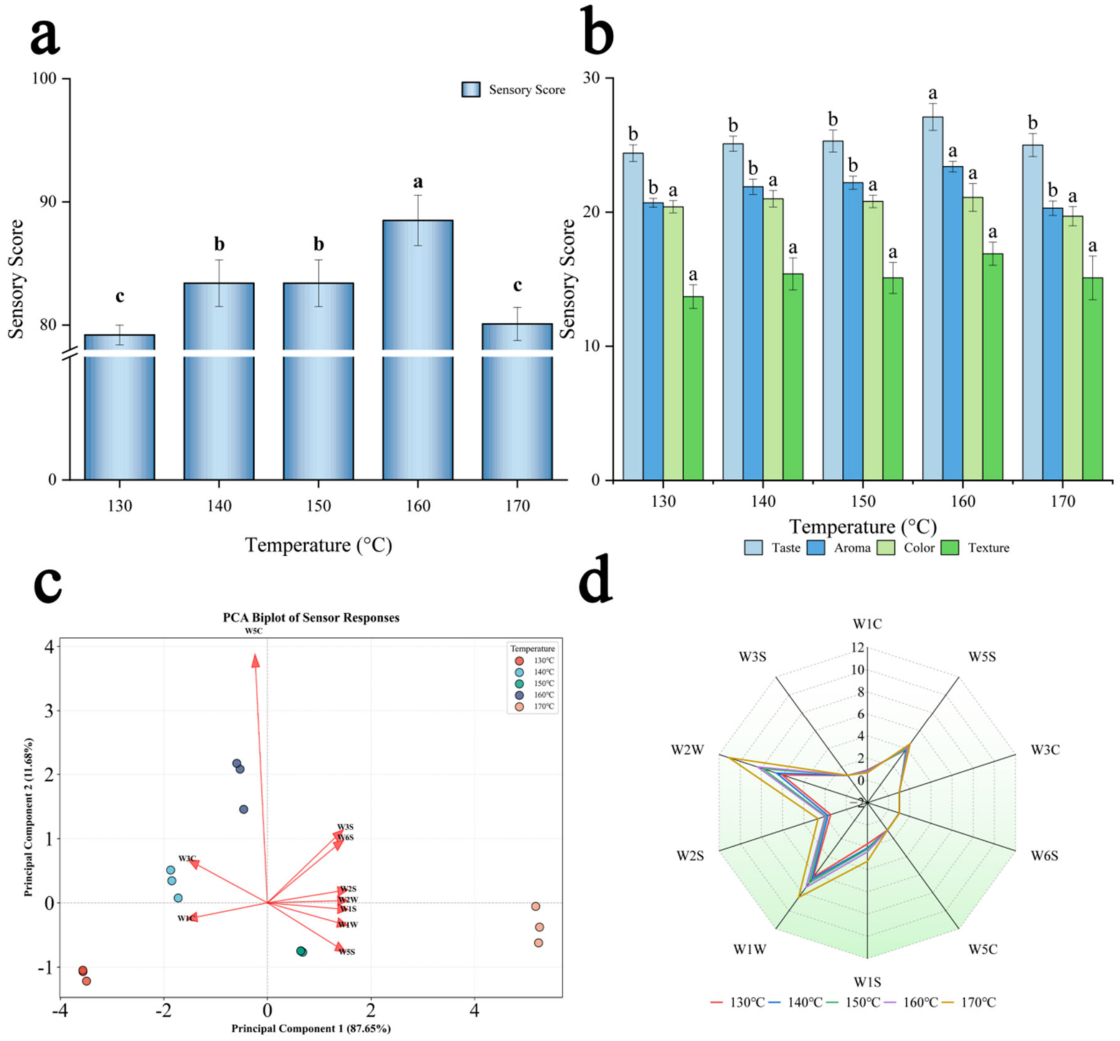

Figure S1. Effects of frying temperature on sensory evaluation and e-nose responses of seasoning oil. (a) overall sensory score; (b) individual sensory attribute scores; (c) e-nose principal component analysis (PCA); (d) e-nose radar plot. Different letters indicate significant differences ($p < 0.05$). Note: W1C, aromatics; W5S, broad-range; W3C, aromatics; W6S, hydrogen; W5C, aromatic/aliphatic hydrocarbons; W1S, broad-methane; W1W, organic sulfur; W2S, alcohols/aldehydes/ketones; W2W, sulfur- and chlorinated compounds; W3S, methane/aliphatic hydrocarbons.

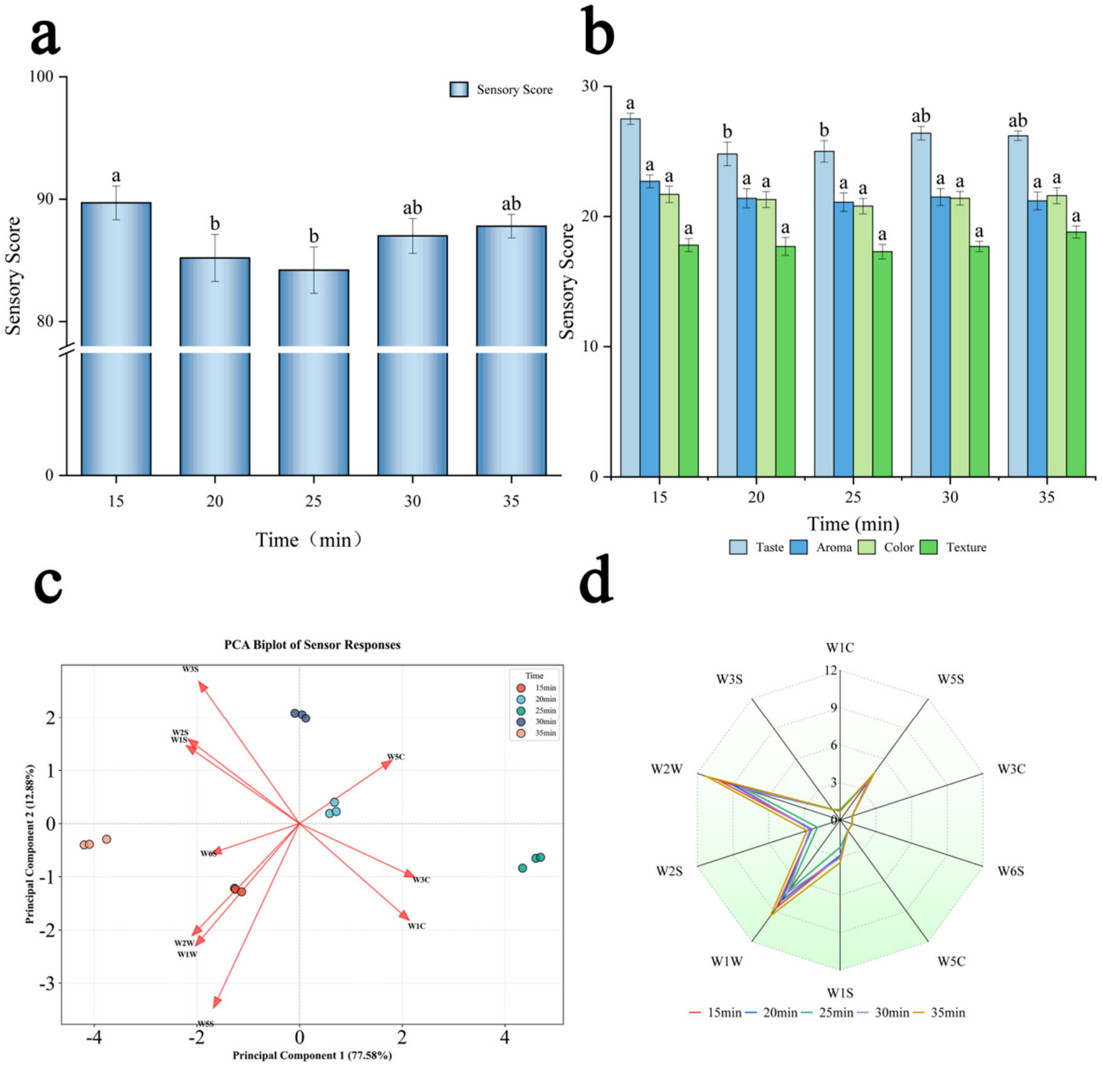

Figure S2. Effects of frying time on sensory evaluation and e-nose responses of seasoning oil. (a) overall sensory score; (b) sensory attribute scores; (c) e-nose principal component analysis (PCA); (d) e-nose radar plot. Different letters indicate significant differences ($p < 0.05$). Note: W1C, aromatics; W5S, broad-range; W3C, aromatics; W6S, hydrogen; W5C, aromatic/aliphatic hydrocarbons; W1S, broad-methane; W1W, organic sulfur; W2S, alcohols/aldehydes/ketones; W2W, sulfur-and chlorinated compounds; W3S, methane/aliphatic hydrocarbons.

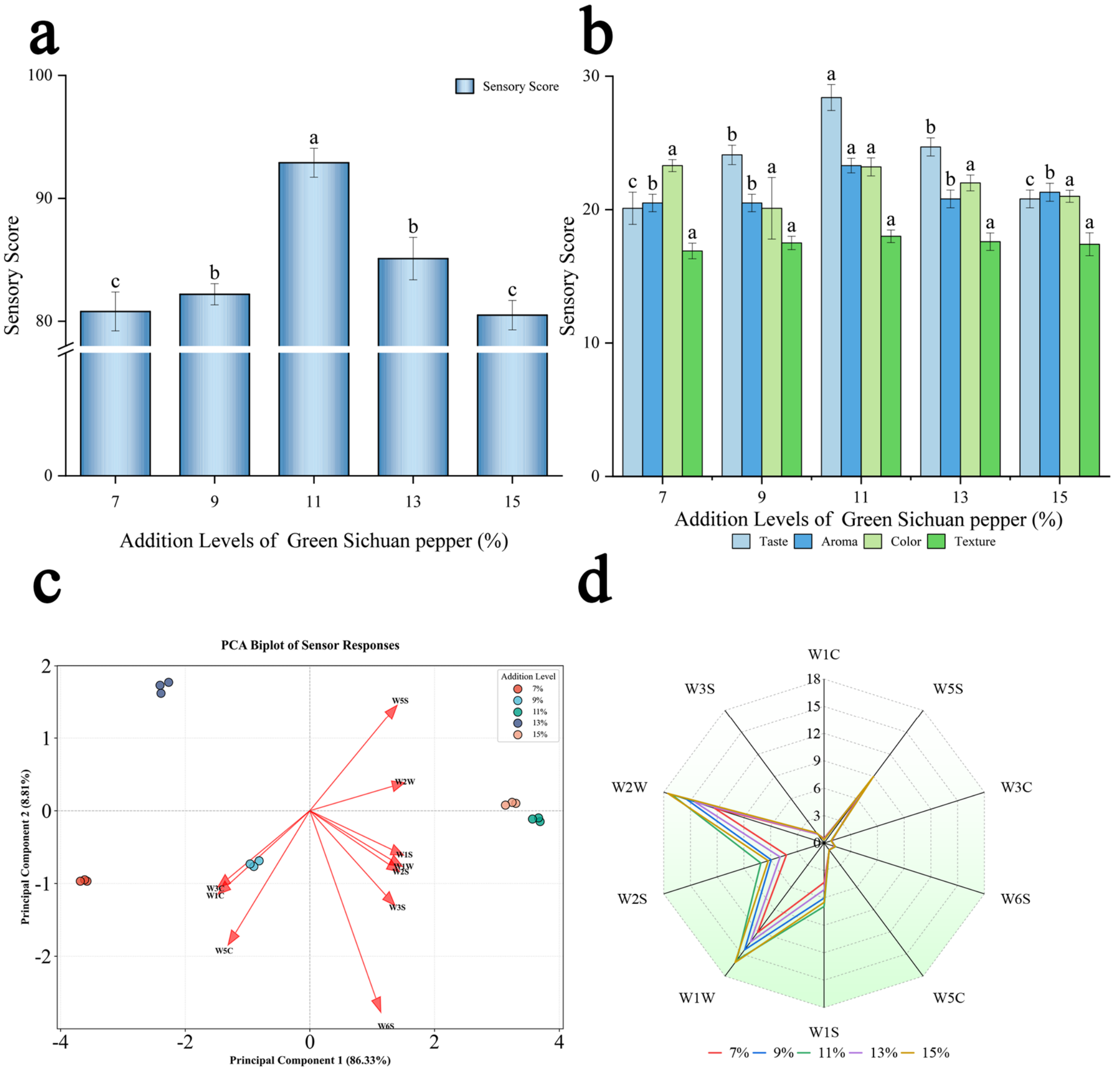

Figure S3. Effects of green Sichuan pepper addition level on sensory evaluation and e-nose responses of seasoning oil. (a) overall sensory score; (b) individual sensory attribute scores; (c) e-nose principal component analysis (PCA); (d) e-nose radar plot. Different letters indicate significant differences ($p < 0.05$). Note: W1C, aromatics; W5S, broad-range; W3C, aromatics; W6S, hydrogen; W5C, aromatic/aliphatic hydrocarbons; W1S, broad-methane; W1W, organic sulfur; W2S, alcohols/aldehydes/ketones; W2W, sulfur and chlorinated compounds; W3S, methane/aliphatic hydrocarbons.

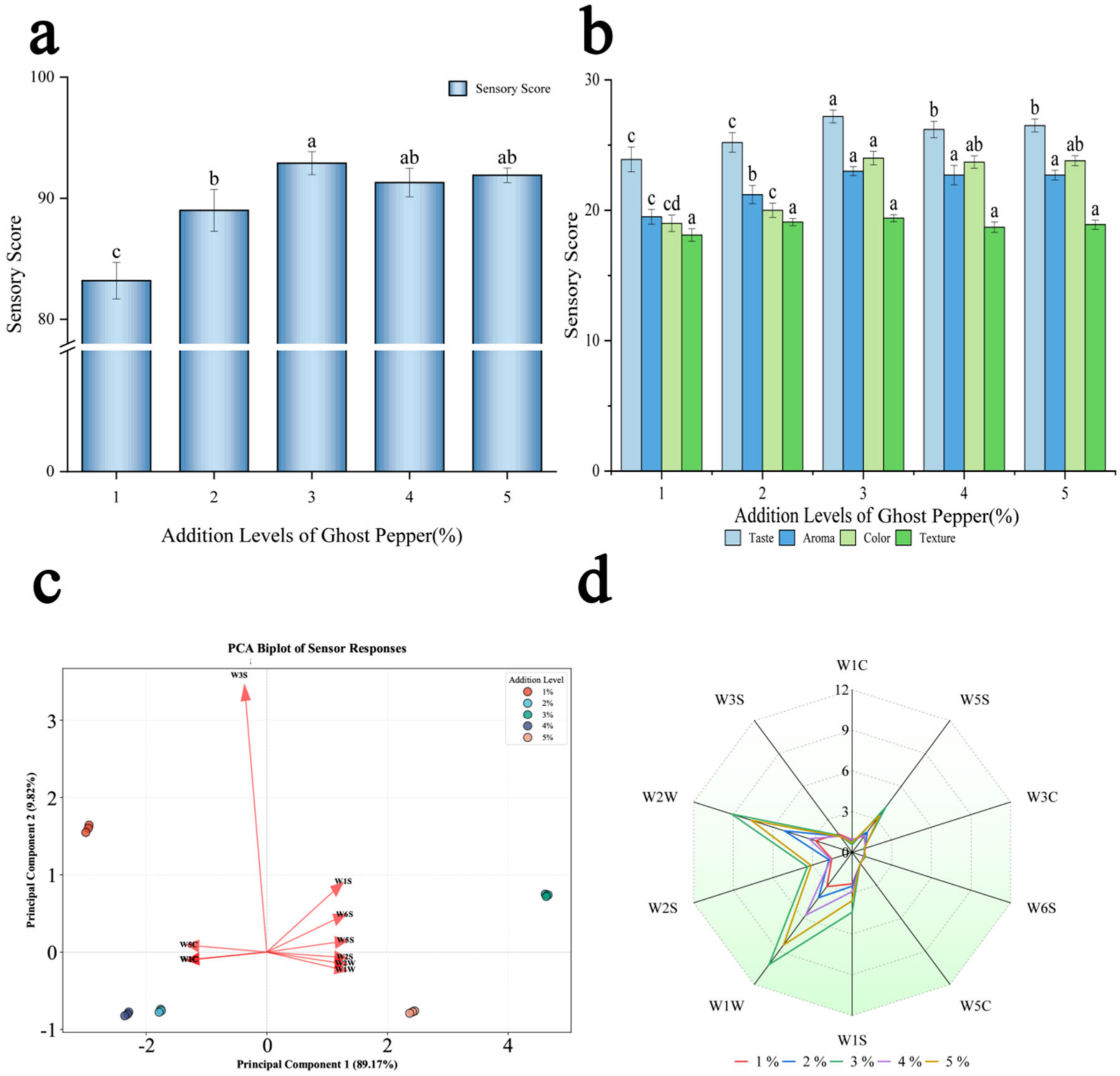

Figure S4. Effects of ghost pepper addition level on sensory evaluation and e-nose responses of seasoning oil. (a) overall sensory score; (b) individual sensory attribute scores; (c) e-nose principal component analysis (PCA); (d) e-nose radar plot. Different letters indicate significant differences ($p < 0.05$). Note: W1C, aromatics; W5S, broad-range; W3C, aromatics; W6S, hydrogen; W5C, aromatic/aliphatic hydrocarbons; W1S, broad-methane; W1W, organic sulfur; W2S, alcohols/aldehydes/ketones; W2W, sulfur and chlorinated compounds; W3S, methane/aliphatic hydrocarbons.

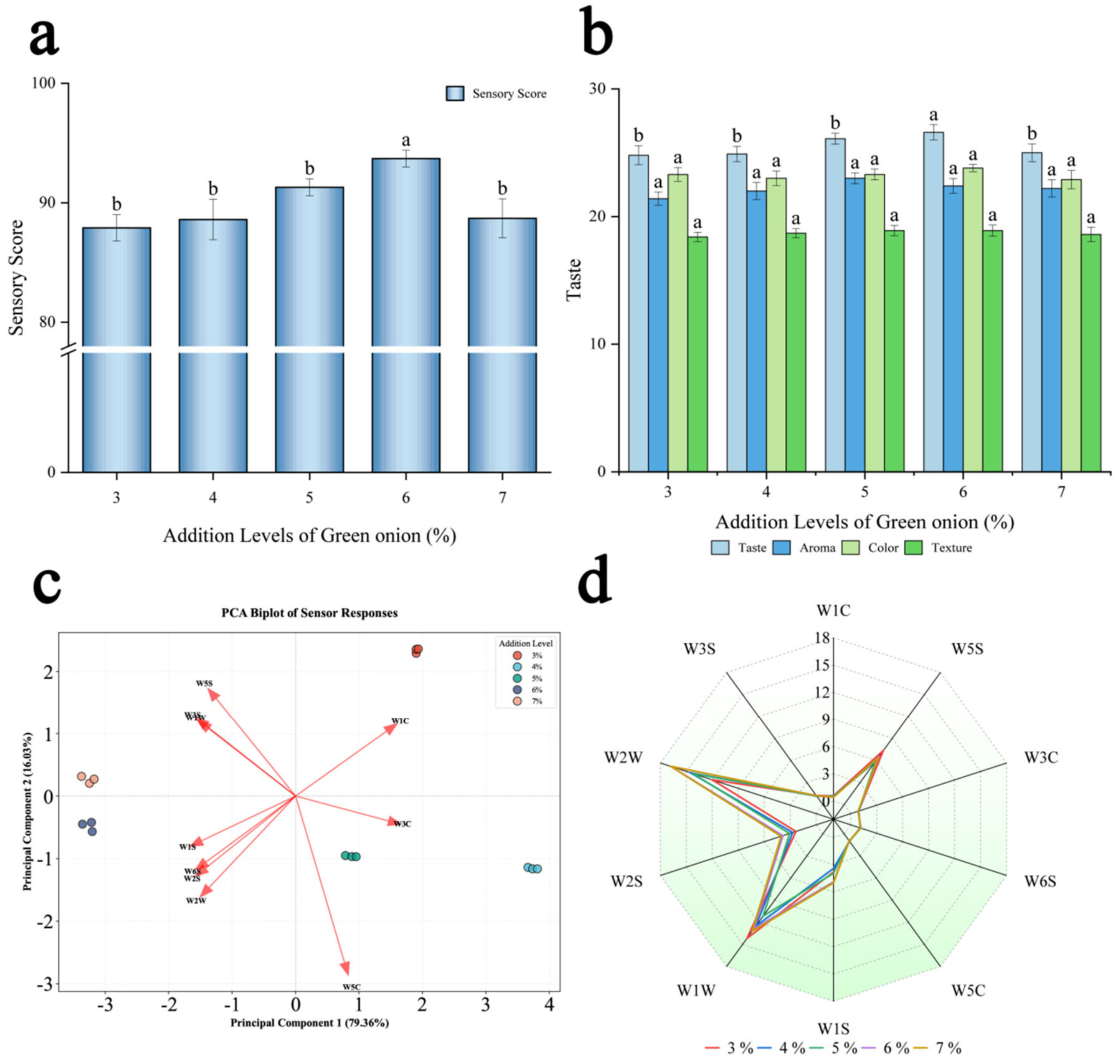

Figure S5. Effects of green onion addition level on sensory evaluation and e-nose responses of seasoning oil. (a) overall sensory score; (b) individual sensory attribute scores; (c) e-nose principal component analysis (PCA); (d) e-nose radar plot. Different letters indicate significant differences ($p < 0.05$). Note: W1C, aromatics; W5S, broad-range; W3C, aromatics; W6S, hydrogen; W5C, aromatic/aliphatic hydrocarbons; W1S, broad-methane; W1W, organic sulfur; W2S, alcohols/aldehydes/ketones; W2W, sulfur and chlorinated compounds; W3S, methane/aliphatic hydrocarbons..

Table S2 Identified volatile components in seasoning oils using gas chromatography–mass spectrometry (GC-MS)

Number	Compounds	Class I	Formula	CAS	Temperature (μg/g)		
					150°C	160°C	170°C
1	Carveol	Terpenoids	$C_{10}H_{16}O$	99-48-9	0.11±0.007	0.136±0.01	0.041±0.04
2	Cyclohexanol, 2-methyl-5-(1-methylethenyl)-	Terpenoids	$C_{10}H_{18}O$	619-01-2	0.273±0.195	0.288±0.195	0.545±0.128
3	2,6-Nonadienal, (E,Z)-	Aldehyde	$C_9H_{14}O$	557-48-2	0.054±0.03	0.104±0.024	0.192±0.093
4	Carvone oxide, cis-	Terpenoids	$C_{10}H_{14}O_2$	18383-49-8	0.129±0.046	0.315±0.041	0.527±0.16
5	Trans-Geranic acid methyl ester	Terpenoids	$C_{11}H_{18}O_2$	1189-09-9	0.03±0.01	0.048±0.017	0.074±0.021
6	endo-fenchol	Alcohol	$C_{10}H_{18}O$	14575-74-7	229.092±26.118	302.21±55.544	284.945±45.649
7	Butanoic acid, 2-methyl-, pentyl ester	Ester	$C_{10}H_{20}O_2$	68039-26-9	0.207±0.11	0.413±0.119	0.36±0.124
8	2-octenal	Aldehyde	$C_8H_{14}O$	2363-89-5	0.043±0.008	0.038±0.011	0.027±0.002
9	Pyrazine, 2-ethyl-3-methyl-	Heterocyclic compound	$C_7H_{10}N_2$	15707-23-0	0.079±0.016	0.113±0.021	0.106±0.015
10	Pyrazine, 2-ethyl-6-methyl-	Heterocyclic compound	$C_7H_{10}N_2$	13925-03-6	0.118±0.06	0.26±0.098	0.216±0.044
11	3-Hexenoic acid, methyl ester, (Z)-	Ester	$C_7H_{12}O_2$	13894-62-7	0.089±0.018	0.128±0.034	0.152±0.025
12	1,3-Octadiene	Hydrocarbons	C_8H_{14}	1002-33-1	0.262±0.277	1.001±0.234	1.38±0.19
13	(+)-(E)-Limonene oxide	Terpenoids	$C_{10}H_{16}O$	6909-30-4	0.038±0.01	0.089±0.028	0.103±0.061
14	3-Furaldehyde	Aldehyde	$C_5H_4O_2$	498-60-2	15.351±5.81	34.504±8.10	42.298±5.08

						8	4
15	2,4,6-Octatriene, 2,6-dimethyl-, (E,Z)-	Terpenoids	$C_{10}H_{16}$	7216-56-0	0.531±0.046	1.229±0.336	1.669±1.103
16	Furan, 2-(1-pentenyl)-, (E)-	Heterocyclic compound	$C_9H_{12}O$	20992-69-2	0.041±0.011	0.059±0.02	0.066±0.003
17	Cyclohexanol, 1-methyl-4-(1-methylethylidene)-, acetate	Ester	$C_{12}H_{20}O_2$	10235-63-9	0.151±0.015	0.251±0.042	0.259±0.05
18	p-Menthane-3,8-diol, cis-1,3,trans-1,4-	Terpenoids	$C_{10}H_{20}O_2$	3564-98-5	0.073±0.116	0.071±0.111	0.139±0.095
19	Geranyl formate	Ester	$C_{11}H_{18}O_2$	105-86-2	0.032±0.048	0.032±0.053	0.065±0.044
20	2-Cyclopenten-1-one, 3-ethyl-2-hydroxy-	Ketone	$C_7H_{10}O_2$	21835-01-8	0.799±0.097	1.077±0.186	0.997±0.126
21	3-Nonen-5-yne, 4-ethyl-, (E)-	Hydrocarbons	$C_{11}H_{18}$	74744-60-8	0.032±0.013	0.1±0.036	0.2±0.159
22	Cis-Muurola-4(15),5-diene	Terpenoids	$C_{15}H_{24}$	157477-72-0	0.083±0.021	0.14±0.037	0.168±0.033
23	1-Butanethiol, 3-methyl-	Alcohol	$C_5H_{12}S$	541-31-1	0.197±0.141	0.566±0.061	0.586±0.115
24	Ethyl tiglate	Ester	$C_7H_{12}O_2$	5837-78-5	0.153±0.116	0.313±0.082	0.365±0.09
25	2(5H)-Furanone	Ketone	$C_4H_4O_2$	497-23-4	0.145±0.117	0.315±0.103	0.274±0.051
26	2(3H)-Furanone	Ketone	$C_4H_4O_2$	20825-71-2	0.728±0.77	3.027±1.312	3.079±0.835
27	2-Butenoic acid, ethyl ester, (Z)-	Ester	$C_6H_{10}O_2$	6776-19-8	0.074±0.04	0.136±0.049	0.159±0.046
28	2-Methylbut-2-en-1-yl acetate	Ester	$C_7H_{12}O_2$	33425-30-8	0.031±0.044	0.113±0.075	0.101±0.051
29	D-Fenchone	Terpenoids	$C_{10}H_{16}O$	4695-62-	91.523±10.1	120.178±21.	113.09±18.0

				9	46	743	52
30	1,6,10-Dodecatrien-3-ol, 3,7,11-trimethyl-, (E)-	Terpenoids	$C_{15}H_{26}O$	40716-66-3	0.126±0.046	0.233±0.058	0.259±0.082
31	Cyclohexanone, 5-methyl-2-(1-methylethyl)-, cis-	Terpenoids	$C_{10}H_{18}O$	491-07-6	0.047±0.036	0.103±0.021	0.098±0.01
32	Benzenamine, N-ethyl-	Amine	$C_8H_{11}N$	103-69-5	0.033±0.002	0.079±0.027	0.112±0.081
33	Fenchone	Terpenoids	$C_{10}H_{16}O$	1195-79-5	91.389±10.179	120±21.751	112.944±18.094
34	1-Octene	Hydrocarbons	C_8H_{16}	111-66-0	0.029±0.008	0.089±0.016	0.088±0.003
35	(+)-4-Carene	Terpenoids	$C_{10}H_{16}$	29050-33-7	0.801±0.356	1.504±0.231	2.187±0.946
36	3-Butylthiophene	Heterocyclic compound	$C_8H_{12}S$	34722-01-5	0.015±0.014	0.084±0.006	0.159±0.124
37	Thiophene, 2,3-dimethyl-	Heterocyclic compound	C_6H_8S	632-16-6	0.056±0.026	0.169±0.111	0.2±0.127
38	3-Hexanone, 2-methyl-	Ketone	$C_7H_{14}O$	2001505	0.21±0.037	0.449±0.045	0.404±0.066
39	2-Decenal, (E)-	Aldehyde	$C_{10}H_{18}O$	3913-81-3	0.041±0.003	0.321±0.112	0.7±0.302
40	Furfural	Aldehyde	$C_5H_4O_2$	35796	16.455±5.974	36.09±8.39	44.039±5.42
41	2,4,6-Octatriene, 2,6-dimethyl-, (E,E)-	Terpenoids	$C_{10}H_{16}$	3016-19-1	0.355±0.068	0.855±0.228	1.206±0.669
42	Dodecane, 4-methyl-	Hydrocarbons	$C_{13}H_{28}$	6117-97-1	0.09±0.019	0.208±0.035	0.272±0.079
43	2,4-Dimethyldodecane	Hydrocarbons	$C_{14}H_{30}$	6117-99-3	0.018±0.005	0.036±0.019	0.055±0.024
44	3-Octanol, 3,7-dimethyl-	Alcohol	$C_{10}H_{22}O$	78-69-3	1.768±0.199	2.326±0.427	2.18±0.347

45	3-Pentenoic acid, 4-methyl-	Acid	$C_6H_{10}O_2$	504-85-8	0.032±0.011	0.051±0.004	0.059±0.01
46	Benzene, 1,3-dimethyl-	Aromatics	C_8H_{10}	108-38-3	1.697±0.945	5.685±2.597	5.722±2.068
47	2-Heptenal, (E)-	Aldehyde	$C_7H_{12}O$	18829-55-5	0.525±0.06	0.895±0.11	0.828±0.268
48	3-Hexenoic acid, methyl ester, (E)-	Ester	$C_7H_{12}O_2$	13894-61-6	0.021±0.004	0.04±0.031	0.085±0.073
49	3-Furanmethanol	Alcohol	$C_5H_6O_2$	4412-91-3	0.168±0.069	0.391±0.096	0.527±0.079
50	2-Octanol, (S)-	Alcohol	$C_8H_{18}O$	1559381	0.207±0.017	0.345±0.039	0.376±0.062
51	1-Hexanol, 4-methyl-	Alcohol	$C_7H_{16}O$	818-49-5	0.918±0.036	1.483±0.132	1.384±0.355
52	Pyrazine, 2,6-dimethyl-	Heterocyclic compound	$C_6H_8N_2$	108-50-9	1.341±1.46	4.085±1.527	3.277±0.772
53	4-Heptenal, (Z)-	Aldehyde	$C_7H_{12}O$	6728-31-0	0.285±0.147	0.617±0.242	0.56±0.119
54	3-Hexen-1-ol, acetate, (Z)-	Ester	$C_8H_{14}O_2$	3681-71-8	0.534±0.227	0.963±0.126	1.351±0.602
55	2-Cyclohexen-1-one, 3-methyl-	Ketone	$C_7H_{10}O$	1193-18-6	0.049±0.016	0.114±0.01	0.292±0.27
56	Acetaldehyde, propylhydrazone	Amine	$C_5H_{12}N_2$	7422-88-0	0.167±0.087	0.358±0.08	0.298±0.036
57	3-Isopropylidene-5-methyl-hex-4-en-2-one	Ketone	$C_{10}H_{16}O$	64149-32-2	0.169±0.069	0.402±0.154	0.293±0.039
58	(R)-(-)-2-Pyrrolidinemethanol	Alcohol	$C_5H_{11}NO$	68832-13-3	0.611±0.4	1.917±0.819	1.75±0.919
59	Fomepizole	Heterocyclic compound	$C_4H_6N_2$	7554-65-6	0.141±0.018	0.225±0.026	0.221±0.064
60	Ethanol, 2-(2-hydroxyethoxy)-, 1-nitrate	Alcohol	$C_4H_9NO_5$	20633-16	0.016±0.011	0.038±0.007	0.042±0.007

				-3			
61	1-Nonene, 4,6,8-trimethyl-	Hydrocarbons	$C_{12}H_{24}$	54410-98-9	0.292±0.036	0.502±0.078	0.591±0.029
62	1,5-Cyclooctadiene, 3,4-dimethyl-	Hydrocarbons	$C_{10}H_{16}$	21284-05-9	0.692±0.267	1.102±0.171	1.57±0.674
63	Benzeneethanamine	Amine	$C_8H_{11}N$	64-04-0	47.963±5.397	63.369±11.619	59.731±9.699
64	2,6-Dimethyldecane	Hydrocarbons	$C_{12}H_{26}$	13150-81-7	0.026±0.006	0.033±0.006	0.042±0.014
65	1,3-Dioxolane, 2-butyl-2-ethyl-	Heterocyclic compound	$C_9H_{18}O_2$	935-49-9	0.034±0.022	0.043±0.03	0.075±0.032
66	2-Acetyl-5-methylfuran	Heterocyclic compound	$C_7H_8O_2$	1193-79-9	0.095±0.111	0.237±0.051	0.225±0.027
67	2H-Pyran, 2-ethenyltetrahydro-2,6,6-trimethyl-	Heterocyclic compound	$C_{10}H_{18}O$	7392-19-0	0.03±0.004	0.036±0.007	0.048±0.006
68	N-Acetylisoxazolidine	Heterocyclic compound	$C_5H_9NO_2$	115615-36-6	0.02±0.014	0.034±0.006	0.01±0.01
69	10-Undecenal	Aldehyde	$C_{11}H_{20}O$	112-45-8	0.08±0.135	0.082±0.131	0.207±0.156
70	Cyclohexanol, 1-ethenyl-	Alcohol	$C_8H_{14}O$	1940-19-8	0.144±0.064	0.263±0.04	0.382±0.165
71	trans-1-Nitro-1-propene	Nitrogen compounds	$C_3H_5NO_2$	17082-05-2	0.097±0.014	0.127±0.023	0.139±0.041
72	Butanoic acid, butyl ester	Ester	$C_8H_{16}O_2$	109-21-7	0.112±0.013	0.16±0.032	0.167±0.021
73	Piperidin-3-one, 2,2,5-methyl-	Ketone	$C_8H_{15}NO$	103634-57-7	0.135±0.104	0.265±0.075	0.26±0.027
74	2-Thiophenecarboxaldehyde, 5-chloro-	Aldehyde	C_5H_3ClOS	7283-96-7	0.581±0.048	5.683±2.338	4.889±1.192

75	Cyclopropanemethanol, 2,2,3,3-tetramethyl-	Alcohol	$C_8H_{16}O$	2415-96-5	0.028±0.02	0.048±0.007	0.065±0.03
76	2-Butenal, 3-methyl-	Aldehyde	C_5H_8O	107-86-8	0.284±0.028	0.503±0.077	0.541±0.113
77	1,4-Pentanediol	Alcohol	$C_5H_{12}O_2$	626-95-9	0.182±0.028	0.329±0.042	0.301±0.048
78	2-Butene-1,4-diol, (Z)-	Alcohol	$C_4H_8O_2$	6117-80-2	0.046±0.004	0.087±0.02	0.092±0.039
79	Butanedioic acid, 2-methyl-3-oxo-, diethyl ester	Ester	$C_9H_{14}O_5$	759-65-9	0.111±0.028	0.317±0.064	0.497±0.175
80	Ethyl 3-cyclohexenecarboxylate	Ester	$C_9H_{14}O_2$	15111-56-5	0.194±0.073	0.234±0.04	0.319±0.085
81	2-Butene, 1-isothiocyanato-	Ester	C_5H_7NS	2253-93-2	0.201±0.033	0.318±0.076	0.306±0.037
82	5-Nonenal, (E)-	Aldehyde	$C_9H_{16}O$	2277-18-1	231.89±26.24	304.403±56.318	287.166±46.081
83	3-Hexene, 1-methoxy-, (Z)-	Ether	$C_7H_{14}O$	70220-06-3	0.102±0.038	0.175±0.015	0.177±0.007
84	Methyl nicotinate	Ester	$C_7H_7NO_2$	93-60-7	0.052±0.014	0.099±0.024	0.102±0.045
85	Urea, N,N'-diethyl-	Amine	$C_5H_{12}N_2O$	623-76-7	0.019±0.014	0.054±0.008	0.039±0.008
86	Alpha.-Aminoisobutanoic acid	Acid	$C_4H_9NO_2$	62-57-7	0.465±0.627	1.205±0.616	0.961±0.423
87	Benzenepropanenitrile	Nitrogen compounds	C_9H_9N	645-59-0	0.038±0.03	0.04±0.007	0.145±0.172
88	2-Ethylhexyl methacrylate	Ester	$C_{12}H_{22}O_2$	688-84-6	0.064±0.11	0.069±0.099	0.132±0.094
89	3-Hexanol, 3-methyl-	Alcohol	$C_7H_{16}O$	597-96-6	0.083±0.008	0.165±0.02	0.171±0.062
90	Butyl carbamate	Ester	$C_5H_{11}NO_2$	592-35-8	0.047±0.002	0.074±0.013	0.082±0.014
91	2-Norbornanone	Ketone	$C_7H_{10}O$	497-38-1	0.093±0.019	0.13±0.025	0.136±0.011
92	2-Butenedioic acid, 2-methyl-, (E)-	Acid	$C_5H_6O_4$	498-24-8	0.111±0.165	0.097±0.157	0.19±0.135
93	Cycloheptanone	Ketone	$C_7H_{12}O$	502-42-1	0.093±0.038	0.15±0.03	0.222±0.096

94	Cycloheptylamine	Amine	$C_7H_{15}N$	5452-35-7	0.199±0.051	0.32±0.046	0.3±0.089
95	Formic acid, octyl ester	Ester	$C_9H_{18}O_2$	112-32-3	1.174±0.321	1.732±0.318	1.965±0.693
96	trans-Ocimenol	Alcohol	$C_{10}H_{18}O$	7643-60-9	0.211±0.049	0.503±0.142	0.676±0.366
97	2-Octyl methylphosphonofluoridate	Ester	$C_9H_{20}FO_2P$	22925-97-9	0.025±0.001	0.047±0.015	0.055±0.021
98	2,5-Furandione, dihydro-3-methyl-	Ketone	$C_5H_6O_3$	4100-80-5	0.034±0.016	0.057±0.012	0.086±0.047
99	Butanoic acid, 2-pentenyl ester, (Z)-	Ester	$C_9H_{16}O_2$	42125-13-3	228.465±25.473	299.11±53.26	281.051±44.674
100	Ethanone, 1-(1H-pyrazol-4-yl)-	Ketone	$C_5H_6N_2O$	25016-16-4	0.024±0.009	0.064±0.035	0.026±0.039
101	3-Nonen-5-one	Ketone	$C_9H_{16}O$	82456-34-6	0.162±0.042	0.34±0.034	0.609±0.399
102	Camphenone, 6-	Ketone	$C_{10}H_{14}O$	55659-42-2	231.747±26.465	304.237±56.002	286.904±45.902
103	Cyclohexene, 2-ethenyl-1,3,3-trimethyl-	Hydrocarbons	$C_{11}H_{18}$	5293-90-3	1.14±0.135	1.542±0.268	1.419±0.19
104	Cyclobutanecarbonitrile, 3,3-dimethyl-	Nitrogen compounds	$C_7H_{11}N$	53783-86-1	0.387±0.188	0.792±0.286	0.729±0.163
105	Maleic anhydride	Acid	$C_4H_2O_3$	108-31-6	0.052±0.015	0.125±0.022	0.184±0.032
106	Methacrylic anhydride	Acid	$C_8H_{10}O_3$	760-93-0	0.015±0.019	0.054±0.023	0.036±0.009
107	Benzene, (butoxymethyl)-	Ether	$C_{11}H_{16}O$	588-67-0	0.043±0.017	0.14±0.081	0.159±0.157
108	[1,1'-Bicyclopentyl]-2-one	Ketone	$C_{10}H_{16}O$	4884-24-6	0.038±0.061	0.047±0.067	0.088±0.065
109	Bis(2-chloro-1-methylethyl) ether	Ether	$C_6H_{12}Cl_2O$	108-60-1	0.033±0.026	0.176±0.107	0.1±0.036

110	2-Furancarboxylic acid, ethyl ester	Ester	$C_7H_8O_3$	614-99-3	0.14±0.027	0.213±0.096	0.286±0.031
111	4-(Methylamino)butyric acid	Acid	$C_5H_{11}NO_2$	1119-48-8	0.179±0.105	0.388±0.107	0.317±0.05
112	Furan, 2-(methoxymethyl)-	Heterocyclic compound	$C_6H_8O_2$	13679-46-4	0.176±0.176	0.936±0.27	0.986±0.078
113	Pentanal, 2,2-dimethyl-	Aldehyde	$C_7H_{14}O$	14250-88-5	0.15±0.147	0.539±0.126	0.738±0.105
114	2-Nonanone, 3-(hydroxymethyl)-	Ketone	$C_{10}H_{20}O_2$	67801-33-6	0.123±0.023	0.173±0.022	0.16±0.006
115	3-Cyclopentyl-1-propanol	Alcohol	$C_8H_{16}O$	767-05-5	0.903±0.066	1.319±0.265	1.284±0.169
116	1H-Imidazole-4-methanol	Alcohol	$C_4H_6N_2O$	822-55-9	0.04±0.01	0.062±0.006	0.053±0.009
117	3,4-Hexanedione	Ketone	$C_6H_{10}O_2$	4437-51-8	0.964±0.134	2.083±0.207	2.054±0.387
118	Decane	Hydrocarbons	$C_{10}H_{22}$	124-18-5	0.202±0.025	0.276±0.035	0.247±0.042
119	5-Heptenal, 2,6-dimethyl-	Aldehyde	$C_9H_{16}O$	106-72-9	0.585±0.078	0.687±0.168	0.763±0.057
120	Heptanoic acid, ethyl ester	Ester	$C_9H_{18}O_2$	106-30-9	0.052±0.006	0.07±0.012	0.064±0.009
121	Cyclohexene, 1-methyl-4-(1-methylethylidene)-	Terpenoids	$C_{10}H_{16}$	586-62-9	230.523±26.18	302.611±56.098	285.76±45.727
122	2,4-Hexadienal, (E,E)-	Aldehyde	C_6H_8O	142-83-6	0.039±0.041	0.135±0.075	0.115±0.046
123	2-Furfurylthiol	Alcohol	C_5H_6OS	35828	0.021±0.018	0.057±0.035	0.056±0.016
124	Pyridine, 2-ethyl-	Heterocyclic compound	C_7H_9N	100-71-0	0.19±0.024	0.266±0.058	0.278±0.052
125	Benzaldehyde	Aldehyde	C_7H_6O	100-52-7	0.341±0.099	0.644±0.172	0.542±0.042
126	2-Furancarboxaldehyde, 5-methyl-	Aldehyde	$C_6H_6O_2$	620-02-0	0.553±0.046	4.696±2.086	8.121±1.003
127	Phenol	Phenol	C_6H_6O	108-95-2	0.138±0.035	0.172±0.019	0.202±0.024
128	2-Thiophenecarboxaldehyde	Aldehyde	C_5H_4OS	35857	0.049±0.018	0.094±0.015	0.135±0.058
129	Pyrazine, trimethyl-	Heterocyclic	$C_7H_{10}N_2$	14667-55	13.495±3.98	21.26±3.834	26.441±10.3

		compound		-1	1		57
130	Hexanoic acid, 3-hydroxy-, ethyl ester	Ester	$C_8H_{16}O_3$	2305-25-1	0.038±0.009	0.067±0.01	0.089±0.033
131	4-Methylthiazole	Heterocyclic compound	C_4H_5NS	693-95-8	0.946±0.394	1.729±0.154	1.811±0.324
132	2(5H)-Furanone, 5-ethyl-	Ketone	$C_6H_8O_2$	2407-43-4	0.031±0.033	0.245±0.074	0.278±0.011
133	Tridecane	Hydrocarbons	$C_{13}H_{28}$	629-50-5	0.114±0.193	0.138±0.197	0.274±0.183
134	2-Undecanone	Ketone	$C_{11}H_{22}O$	112-12-9	1.621±2.541	1.905±2.58	3.426±2.318
135	Pyridine, 2-pentyl-	Heterocyclic compound	$C_{10}H_{15}N$	2294-76-0	0.096±0.05	0.141±0.049	0.206±0.056
136	1-Cyclohexene-1-carboxaldehyde, 4-(1-methylethenyl)-	Aldehyde	$C_{10}H_{14}O$	2111-75-3	0.092±0.035	0.169±0.099	0.058±0.018
137	Ethanone, 1-(2-furanyl)-	Ketone	$C_6H_6O_2$	1192-62-7	0.275±0.298	1.026±0.419	0.759±0.102
138	Pyrazine, 2-methoxy-3-methyl-	Heterocyclic compound	$C_6H_8N_2O$	2847-30-5	0.049±0.004	0.431±0.179	0.535±0.064
139	3-Methoxy-5-methylphenol	Phenol	$C_8H_{10}O_2$	3209-13-0	0.02±0.003	0.042±0.022	0.047±0.021
140	1-Butene, 4-isothiocyanato-	Ester	C_5H_7NS	3386-97-8	0.236±0.048	0.235±0.052	0.104±0.044
141	Bicyclo[3.1.1]heptan-3-one, 2-hydroxy-2,6,6-trimethyl-	Ketone	$C_{10}H_{16}O_2$	10136-65-9	0.005±0	0.038±0.009	0.064±0.022
142	1-Octyne	Hydrocarbons	C_8H_{14}	629-05-0	0.032±0.014	0.114±0.031	0.122±0.013
143	2-Isopropyl-5-methylhex-2-enal	Aldehyde	$C_{10}H_{18}O$	35158-25-9	8.491±0.987	11.17±2.071	10.527±1.643
144	3-Decen-2-one	Ketone	$C_{10}H_{18}O$	10519-33	0.103±0.006	0.142±0.012	0.131±0.01

				-2			
145	1-Octanol, 3,7-dimethyl-	Alcohol	$C_{10}H_{22}O$	106-21-8	0.258±0.185	0.385±0.132	0.525±0.201
146	Octanal, 7-hydroxy-3,7-dimethyl-	Aldehyde	$C_{10}H_{20}O_2$	107-75-5	0.044±0.075	0.058±0.077	0.099±0.067
147	3-Hepten-2-one	Ketone	$C_7H_{12}O$	1119-44-4	0.022±0.011	0.038±0.011	0.047±0.006
148	9-Decen-1-ol	Alcohol	$C_{10}H_{20}O$	13019-22-2	0.06±0.019	0.192±0.003	0.257±0.125
149	Pyrazine, methyl-	Heterocyclic compound	$C_5H_6N_2$	109-08-0	0.934±0.412	1.779±0.861	1.691±0.39
150	7-Octen-4-ol, 2-methyl-6-methylene-, (S)-	Terpenoids	$C_{10}H_{18}O$	35628-05-8	28.726±3.117	37.537±6.769	35.243±5.544
151	4-Methylpentyl 2-methylbutanoate	Ester	$C_{11}H_{22}O_2$	35852-40-5	0.064±0.021	0.102±0.017	0.096±0.007
152	1-Hexanol, 5-methyl-2-(1-methylethyl)-, acetate	Ester	$C_{12}H_{24}O_2$	40853-55-2	0.218±0.035	0.596±0.064	0.814±0.357
153	Undecanol-4	Alcohol	$C_{11}H_{24}O$	866512	0.039±0.066	0.048±0.079	0.101±0.068
154	Cyclohexanone, 4-methyl-	Ketone	$C_7H_{12}O$	589-92-4	1.03±0.045	1.694±0.167	1.658±0.468
155	2(5H)-Furanone, 5,5-dimethyl-	Ketone	$C_6H_8O_2$	20019-64-1	0.094±0.015	0.192±0.017	0.193±0.063
156	2,4-Hexadien-1-ol	Alcohol	$C_6H_{10}O$	111-28-4	0.019±0.009	0.034±0.013	0.045±0.007
157	Cyclohexane, propyl-	Hydrocarbons	C_9H_{18}	1678-92-8	0.027±0.014	0.043±0.008	0.058±0.025
158	2-Cyclopenten-1-one, 2-hydroxy-	Ketone	$C_5H_6O_2$	10493-98-8	0.137±0.007	0.26±0.063	0.273±0.126
159	Cyclohexanol, 2-(1,1-dimethylethyl)-, acetate, cis-	Ester	$C_{12}H_{22}O_2$	20298-69-5	0.076±0.13	0.083±0.119	0.162±0.108
160	2,4-Heptadienal, (E,E)-	Aldehyde	$C_7H_{10}O$	881395	13.813±3.96	21.532±3.88	26.749±10.5

					5		12
161	1,5-Pentanediamine	Amine	$C_5H_{14}N_2$	462-94-2	0.164±0.028	0.232±0.01	0.248±0.047
162	Lactic acid	Acid	$C_3H_6O_3$	50-21-5	0.062±0.015	0.086±0.016	0.105±0.016
163	3-Pentanol, 2-methyl-	Alcohol	$C_6H_{14}O$	565-67-3	0.196±0.032	0.249±0.055	0.295±0.033
164	Urea	Nitrogen compounds	CH_4N_2O	57-13-6	0.017±0.018	0.058±0.008	0.077±0.028
165	2(5H)-Furanone, 5-methyl-	Ketone	$C_5H_6O_2$	591-11-7	0.176±0.004	0.302±0.038	0.267±0.073
166	N-Ethylmorpholine	Heterocyclic compound	$C_6H_{13}NO$	100-74-3	1.048±0.553	3.612±1.657	3.665±1.353
167	2(3H)-Furanone, dihydro-5-methyl-	Ketone	$C_5H_8O_2$	108-29-2	0.064±0.003	0.115±0.008	0.093±0.006
168	Pentanoic acid, butyl ester	Ester	$C_9H_{18}O_2$	591-68-4	16.416±1.795	21.322±3.797	19.941±3.035
169	2H-Pyran-2-one, tetrahydro-6-methyl-	Ketone	$C_6H_{10}O_2$	823-22-3	13.181±1.463	17.296±3.166	16.304±2.636
170	3-Pentanol, 2,3,4-trimethyl-	Alcohol	$C_8H_{18}O$	3054-92-0	0.039±0.021	0.085±0.033	0.081±0.014
171	Tricyclo[2.2.1.0(2,6)]heptane, 1,7,7-trimethyl-	Terpenoids	$C_{10}H_{16}$	508-32-7	0.038±0.003	0.068±0.018	0.07±0.034
172	2-Dimethylamino-4-methyl-pent-4-enenitrile	Amine	$C_8H_{14}N_2$	94492-10-1	49.566±5.557	64.736±12.164	61.4±9.79
173	6-Nonen-1-ol, acetate, (Z)-	Ester	$C_{11}H_{20}O_2$	76238-22-7	0.039±0.01	0.062±0.014	0.077±0.034
174	1-Propanone, 1-phenyl-	Ketone	$C_9H_{10}O$	93-55-0	0.036±0.027	0.099±0.02	0.111±0.024
175	3-Hexanol, 5-methyl-	Alcohol	$C_7H_{16}O$	623-55-2	0.044±0.02	0.134±0.035	0.173±0.03
176	Propanamide	Amine	C_3H_7NO	79-05-0	0.048±0.032	0.103±0.03	0.085±0.013
177	5-Hepten-2-ol, 6-methyl-	Alcohol	$C_8H_{16}O$	1569-60-4	0.107±0.018	0.036±0.056	0.097±0.017
178	1-Tridecyne	Hydrocarbons	$C_{13}H_{24}$	26186-02	0.055±0.092	0.063±0.084	0.111±0.078

				-7			
179	Furan, 3-(4,8-dimethyl-3,7-nonadienyl)-, (E)-	Terpenoids	$C_{15}H_{22}O$	23262-34-2	0.175±0.07	0.308±0.053	0.316±0.101
180	Cyclopentanone	Ketone	C_5H_8O	120-92-3	0.112±0.02	0.287±0.051	0.282±0.091
181	Cyclohexene, 4-ethenyl-	Hydrocarbons	C_8H_{12}	100-40-3	0.361±0.142	0.781±0.167	0.777±0.09
182	Ethanol, 2-butoxy-	Alcohol	$C_6H_{14}O_2$	111-76-2	0.073±0.036	0.176±0.085	0.156±0.047
183	Pyrrolidine, 1-acetyl-	Heterocyclic compound	$C_6H_{11}NO$	4030-18-6	0.048±0.006	0.065±0.01	0.063±0.005
184	1,2-Cyclopentanedione, 3-methyl-	Ketone	$C_6H_8O_2$	765-70-8	1.601±0.992	3.245±0.634	3.155±0.611
185	2-Methyl-6-methyleneocta-2,7-dien-4-one	Terpenoids	$C_{10}H_{14}O$	539-70-8	0.087±0.111	0.256±0.07	0.162±0.058
186	Ascaridole	Terpenoids	$C_{10}H_{16}O_2$	512-85-6	0.094±0.031	0.138±0.013	0.172±0.062
187	Phenol, 2-(1,1-dimethylethyl)-	Phenol	$C_{10}H_{14}O$	88-18-6	0.085±0.131	0.193±0.036	0.126±0.048
188	3-Methylthiobutyraldehyde	Aldehyde	$C_5H_{10}OS$	16630-52-7	0.738±0.081	1.225±0.136	1.153±0.319
189	Thiazole, 2,4-diethyl-	Heterocyclic compound	$C_7H_{11}NS$	32272-49-4	0.043±0.027	0.135±0.039	0.124±0.019
190	Furfuryl formate	Ester	$C_6H_6O_3$	13493-97-5	0.019±0.013	0.051±0.005	0.044±0.009
191	Butanedioic acid	Acid	$C_4H_6O_4$	110-15-6	0.02±0.018	0.099±0.08	0.203±0.08
192	1-Nonen-3-ol	Alcohol	$C_9H_{18}O$	21964-44-3	0.061±0.102	0.22±0.183	0.21±0.085
193	Hexyl tiglate	Ester	$C_{11}H_{20}O_2$	16930-96-4	0.286±0.139	0.553±0.124	0.633±0.08
194	Pyrazine, 2-(n-propyl)-	Heterocyclic compound	$C_7H_{10}N_2$	18138-03-9	0.303±0.021	0.713±0.267	1.097±0.611
195	1-(1,3-Oxazol-2-yl)ethan-1-one	Ketone	$C_5H_5NO_2$	77311-07-0	0.062±0.039	0.159±0.104	0.16±0.035

196	Butanoic acid, 3-oxo-, butyl ester	Ester	$C_8H_{14}O_3$	591-60-6	15.16±1.703	19.834±3.616	18.724±2.871
197	(Z)-Hex-3-enyl (E)-2-methylbut-2-enoate	Ester	$C_{11}H_{18}O_2$	67883-79-8	1.739±1.274	4.041±1.048	5.933±2.552
198	(-)-trans-Isopiperitenol	Terpenoids	$C_{10}H_{16}O$	74410-00-7	0.11±0.007	0.136±0.01	0.041±0.04
199	trans-Carveol	Terpenoids	$C_{10}H_{16}O$	1197-07-5	0.11±0.007	0.136±0.01	0.041±0.04
200	(-)-cis-Isopiperitenol	Terpenoids	$C_{10}H_{16}O$	96555-02-1	0.11±0.007	0.136±0.01	0.041±0.04
201	Cyclohexanol, 1-methyl-4-(1-methylethylidene)-	Terpenoids	$C_{10}H_{18}O$	586-81-2	0.273±0.195	0.288±0.195	0.545±0.128
202	2,6-Octadienoic acid, 3,7-dimethyl-, methyl ester	Ester	$C_{11}H_{18}O_2$	2349-14-6	0.03±0.01	0.048±0.017	0.074±0.021
203	Dodecane, 3-methyl-	Hydrocarbons	$C_{13}H_{28}$	17312-57-1	0.09±0.019	0.208±0.035	0.272±0.079
204	Fenchol	Terpenoids	$C_{10}H_{18}O$	1632-73-1	229.092±26.118	302.21±55.544	284.945±45.649
205	Pentanoic acid, pentyl ester	Ester	$C_{10}H_{20}O_2$	2173-56-0	0.207±0.11	0.413±0.119	0.36±0.124
206	Pyrazine, 2-ethyl-5-methyl-	Heterocyclic compound	$C_7H_{10}N_2$	13360-64-0	0.079±0.016	0.113±0.021	0.106±0.015
207	(+)-3-Carene	Terpenoids	$C_{10}H_{16}$	498-15-7	0.801±0.356	1.504±0.231	2.187±0.946
208	2,4-Octadiene	Hydrocarbons	C_8H_{14}	13643-08-8	0.262±0.277	1.001±0.234	1.38±0.19
209	2,4,6-Octatriene, 2,6-dimethyl-	Terpenoids	$C_{10}H_{16}$	673-84-7	0.531±0.046	1.229±0.336	1.669±1.103
210	cis-2-(2-Pentenyl)furan	Heterocyclic	$C_9H_{12}O$	70424-13	0.041±0.011	0.059±0.02	0.066±0.003

		compound		-4			
211	(1Alpha,3beta,4beta)-p-menthane-3,8-diol	Terpenoids	$C_{10}H_{20}O_2$	3564-95-2	0.073±0.116	0.071±0.111	0.139±0.095
212	2,6-Octadien-1-ol, 3,7-dimethyl-, formate, (Z)-	Ester	$C_{11}H_{18}O_2$	2142-94-1	0.032±0.048	0.032±0.053	0.065±0.044
213	2-Hydroxy-3,5-dimethylcyclopent-2-en-1-one	Ketone	$C_7H_{10}O_2$	21834-98-0	0.799±0.097	1.077±0.186	0.997±0.126
214	3,4-Dimethyl-1,2-cyclopentadione	Ketone	$C_7H_{10}O_2$	13494-06-9	0.799±0.097	1.077±0.186	0.997±0.126
215	3-Nonen-5-yne, 4-ethyl-, (Z)-	Hydrocarbons	$C_{11}H_{18}$	74744-26-6	0.032±0.013	0.1±0.036	0.2±0.159
216	(1S,4S,4aS)-1-Isopropyl-4,7-dimethyl-1,2,3,4,4a,5-hexahydronaphthalene	Terpenoids	$C_{15}H_{24}$	267665-20-3	0.083±0.021	0.14±0.037	0.168±0.033
217	1-Butanethiol, 2-methyl-	Alcohol	$C_5H_{12}S$	1878-18-8	0.197±0.141	0.566±0.061	0.586±0.115
218	2-Butenoic acid, ethyl ester, (E)-	Ester	$C_6H_{10}O_2$	623-70-1	0.074±0.04	0.136±0.049	0.159±0.046
219	2-Buten-1-ol, 3-methyl-, acetate	Ester	$C_7H_{12}O_2$	1191-16-8	0.031±0.044	0.113±0.075	0.101±0.051
220	L-Fenchone	Terpenoids	$C_{10}H_{16}O$	7787-20-4	91.523±10.146	120.178±21.743	113.09±18.052
221	Nerolidol	Terpenoids	$C_{15}H_{26}O$	142-50-7	0.126±0.046	0.233±0.058	0.259±0.082
222	Cis-Nerolidol	Terpenoids	$C_{15}H_{26}O$	3790-78-1	0.126±0.046	0.233±0.058	0.259±0.082
223	2-Octene	Hydrocarbons	C_8H_{16}	111-67-1	0.029±0.008	0.089±0.016	0.088±0.003
224	Thiophene, 2,4-dimethyl-	Heterocyclic compound	C_6H_8S	638-00-6	0.056±0.026	0.169±0.111	0.2±0.127
225	Thiophene, 2,5-dimethyl-	Heterocyclic	C_6H_8S	638-02-8	0.056±0.026	0.169±0.111	0.2±0.127

		compound					
226	Thiophene, 3,4-dimethyl-	Heterocyclic compound	C_6H_8S	632-15-5	0.056±0.026	0.169±0.111	0.2±0.127
227	3-Pentanone, 2,4-dimethyl-	Ketone	$C_7H_{14}O$	565-80-0	0.21±0.037	0.449±0.045	0.404±0.066
228	3,5-Dimethyldodecane	Hydrocarbons	$C_{14}H_{30}$	107770-99-0	0.018±0.005	0.036±0.019	0.055±0.024
229	Dodecane, 4,6-dimethyl-	Hydrocarbons	$C_{14}H_{30}$	61141-72-8	0.018±0.005	0.036±0.019	0.055±0.024
230	3-Octanol, 3,6-dimethyl-	Alcohol	$C_{10}H_{22}O$	151-19-9	1.768±0.199	2.326±0.427	2.18±0.347
231	2-Pentenoic acid, 4-methyl-	Acid	$C_6H_{10}O_2$	10321-71-8	0.032±0.011	0.051±0.004	0.059±0.01
232	Ethylbenzene	Aromatics	C_8H_{10}	100-41-4	1.697±0.945	5.685±2.597	5.722±2.068
233	p-Xylene	Aromatics	C_8H_{10}	106-42-3	1.697±0.945	5.685±2.597	5.722±2.068
234	2-Heptenal, (Z)-	Aldehyde	$C_7H_{12}O$	57266-86-1	0.525±0.06	0.895±0.11	0.828±0.268
235	2-Furanmethanol	Alcohol	$C_5H_6O_2$	98-00-0	0.168±0.069	0.391±0.096	0.527±0.079
236	2-Octanol	Alcohol	$C_8H_{18}O$	123-96-6	0.207±0.017	0.345±0.039	0.376±0.062
237	1-Hexanol, 5-methyl-	Alcohol	$C_7H_{16}O$	627-98-5	0.918±0.036	1.483±0.132	1.384±0.355
238	1-Hexanol, 4-methyl-, (S)-	Alcohol	$C_7H_{16}O$	1767-46-0	0.918±0.036	1.483±0.132	1.384±0.355
239	Pyrazine, 2,5-dimethyl-	Heterocyclic compound	$C_6H_8N_2$	123-32-0	1.341±1.46	4.085±1.527	3.277±0.772
